# Striatal Nitric Oxide Activity Better Predicts Motor Disability Than Proto‐Oncogenes

**DOI:** 10.1111/ejn.70386

**Published:** 2026-01-13

**Authors:** Sonia Guerrero Prieto, Victor Ricardo C. Torres da Silva, Maria Camila Almeida, Marcela B. Echeverry

**Affiliations:** ^1^ Graduate Program in Neuroscience & Cognition, Center for Mathematics, Computation and Cognition Universidade Federal do ABC São Bernardo do Campo São Paulo Brazil; ^2^ Center for Natural and Human Sciences (CCNH) Universidade Federal do ABC São Bernardo do Campo São Paulo Brazil; ^3^ Center for Mathematics, Computation and Cognition Universidade Federal do ABC São Bernardo do Campo São Paulo Brazil; ^4^ Neuroscience Laboratory—School of Medicine Universidad de Santander (UDES) Bucaramanga Santander Colombia

**Keywords:** catalepsy, haloperidol, metoclopramide, nitric oxide synthase, nucleus accumbens

## Abstract

Extrapyramidal symptoms (EPS) are side effects observed after acute administration of D2 antagonists and nitric oxide synthase (NOS) inhibitors in rodents. To date, no study has examined NOS activity in parallel with c‐Fos immunoreactivity (c‐Fos‐IR) following multiple doses of these compounds. The aim of the present study was to evaluate whether catalepsy and motor balance deficits resulting from specific acute doses of haloperidol (Hal), metoclopramide (MCP), and L‐NOARG could correlate with changes in the number of c‐Fos‐IR and nNOS‐positive cells, as well as NADPH‐diaphorase activity in the striatum. Male Swiss mice received Hal (0.1–1 mg/kg, ip), MCP (1–45 mg/kg, ip), L‐NOARG (15–45 mg/kg, ip), or saline. An increased cataleptic effect was observed in all experimental groups. All doses of Hal and the higher doses of MCP resulted in deficits in the Rota‐rod test, whereas L‐NOARG did not affect Rota‐rod performance. Histochemical analysis revealed increased c‐Fos‐IR in the dorsal striatum following Hal, as well as in the dorsolateral striatum after low and intermediate clinically relevant doses of MCP. Both types of D2R antagonists led to an increase in NADPH‐diaphorase activity in the dorsal striatum. Similarly, the higher catalepsy‐inducing doses of L‐NOARG resulted in increased NADPH‐diaphorase activity in the dorsal striatum; however, these same doses also reduced c‐Fos‐IR in the dorsolateral striatum and nucleus accumbens (NAc). In conclusion, all drugs acutely induced catalepsy, with motor balance preserved after L‐NOARG treatment. Our findings suggest that EPS side effects may be attributed to NADPH‐diaphorase activity in the dorsal striatum.

## Graphical abstract

The figure summarizes the experimental design in which mice received a single intraperitoneal injection of saline, haloperidol, metoclopramide, or L‐NOARG at different doses. Extrapyramidal (EPS) side effects were assessed using the catalepsy test and the Rota‐rod test. All drugs induced catalepsy and reduced motor performance, except for L‐NOARG. Acute EPS side effects were significantly correlated with NADPH‐d activity in the dorsal striatum for catalepsy and with nNOS/NADPH‐d activity in the dorsomedial striatum for Rota‐rod performance.

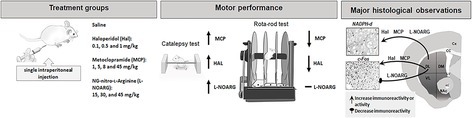

**Abbreviations**
7‐NI: 7‐nitroindazole
c‐Fos‐IR: c‐Fos immunoreactivity
D2Rs: dopamine D2 receptors
DA: dopamine
DL: striatal dorsolateral area
DM: striatal dorsomedial area
EPS: extrapyramidal symptoms
Hal: haloperidol
L‐NOARG: N^ω^‐nitro‐l‐arginine (a l‐arginine‐reversible inhibitor)
MCP: metoclopramide
MSNs: medium spiny projection neurons
NAc: nucleus accumbens
NADPH: nicotinamide adenine dinucleotide phosphate
NADPH‐d: nicotinamide adenine dinucleotide phosphate diaphorase
NMDA: N‐methyl‐D‐aspartate receptor (glutamatergic)
nNOS/NOS1: neuronal nitric oxide synthase
NO: nitric oxide
NOS: nitric oxide synthase
VL: striatal ventrolateral area

## 1 Introduction

The striatum serves as an integrative motor hub, where coordinated interactions among glutamate, nitric oxide (NO), and dopamine (DA) critically shape the activity of striatal microcircuits. These neurotransmitter systems converge onto nitric oxide synthase (NOS) interneurons and medium spiny neurons (MSNs) (Kiss and Vizi 2001; Sammut et al. 2007), and both corticostriatal glutamatergic and nigrostriatal dopaminergic projections target striatal GABAergic (Mallet et al. 2005) and nitrergic interneurons (Sammut et al. 2007; Vuillet et al. 1989). Through these pathways, NO functions as both a retrograde messenger and a modulator of postsynaptic plasticity, particularly by facilitating long‐term depression (LTD) in MSNs (Calabresi et al. 1999, 2014). Although motor control within the basal ganglia primarily depends on the activation of cortical networks that drive direct (striatonigral) and indirect (striatopallidal) pathways (DeLong and Wichmann 2007), alterations in the synaptic plasticity of striatal microcircuits can disrupt this coordination, contributing to persistent extrapyramidal side effects (EPS) and dyskinesia (Bonito‐Oliva et al. 2011).

Extrapyramidal symptoms have been associated not only with various antipsychotic drugs but also with metoclopramide (MCP) (Agovic et al. 2008), an antiemetic commonly used in clinical care (Miller and Jankovic 1989). These symptoms have also been reported in preclinical studies following acute systemic (Del‐Bel et al. 2010; Marras et al. 1995), intracerebroventricular (Echeverry et al. 2007), and intrastriatal (E. A. Del Bel et al. 2004) administration of NOS inhibitors. Frequently, the effects of NOS inhibitors are examined with NADPH‐diaphorase (NADPH‐d) activity. Previously, the NADPH‐d reaction has been used to monitor NOS activity in fixed tissue (Matsumoto et al. 1993). Therefore, NADPH‐d histochemistry provides a specific marker for neurons producing NO (Dawson et al. 1991; Hope et al. 1991).

Both D2 receptor antagonism and NOS inhibition are known to induce cataleptic effects, providing a rationale for comparing haloperidol (Hal), MCP, and NOS inhibitors in the present study. Highlighting the link between D2R blockade and NO signaling, early work with the D2R antagonist eticlopride demonstrated increased NOS activity (Morris et al. 1997) and facilitated NO efflux in the striatum (Sammut et al. 2007). Chronic treatment with a cataleptic dose of Hal in the context of tardive dyskinesia models increased NOS activity, nNOS protein, and nNOS mRNA expression in rat striatum, but not in the NAc (Lau et al. 2003). In contrast, Hal appears to inhibit neuronal NOS activity in vitro (Hu et al. 1994). In another study, subchronic—but not acute—L‐NOARG treatment increased the number of NADPH‐d positive neurons in the dorsal striatum and NAc compared with Hal. Both L‐NOARG and Hal, however, decreased NADPH‐d‐positive neurons in the substantia nigra pars compacta (Del‐Bel and Guimarães 2000). To date, MCP side effects have not been associated with NOS/NADPH‐d activity in the striatum.

In light of these findings, MCP appears to act as a D2R antagonist at nanomolar doses, in addition to being a mixed 5‐HT_3_ receptor antagonist/5‐HT_4_ receptor agonist, in the chemoreceptor trigger zone located in the central nervous system (Al‐Saffar et al. 2019). However, the neuropharmacology of MCP is complex, and the role of D2R occupancy in its use is not well established, in contrast to the clearly defined role of D2R occupancy in the motor symptoms induced by Hal, a well‐known typical antipsychotic. Antipsychotic‐induced catalepsy has been associated with DA‐D2R occupancy in the nucleus accumbens (NAc) (Hartgraves and Kelly 1984) and the neostriatum (Dunstan and Broekkamp 1981), with activation of the dorsolateral striatum (DL) for Hal (Sonego et al. 2016) and dorsomedial striatum (DM) for atypical antipsychotic drugs (Batista et al. 2016).

The previous statement is because proto‐oncogene c‐Fos has been widely used to investigate EPS‐induced activation of the striatum. Typical antipsychotics increase Fos protein staining in DL striatum, whereas other antipsychotics, such as those of the atypical class, enhance c‐Fos immunoreactivity (c‐Fos‐IR) in the NAc shell region—a finding widely accepted in the scientific literature (Merchant and Dorsa 1993; Robertson et al. 1994; Sebens et al. 1995). In fact, EPS liability has been linked to striatal matrix, as typical antipsychotics induce a pronounced c‐Fos‐IR in this compartment (Bubser and Deutch 2002), where NOS‐expressing GABAergic interneurons are also primarily located (Brimblecombe and Cragg 2017), positioned between matrix‐striosome compartment borders and participating in the striatal network (Saka et al. 2002).

As for MCP, the c‐Fos‐IR induced by MDMA (3,4‐Methylenedioxymethamphetamine)—a drug of abuse with significant effects in the striatum was inhibited by MCP in isolated dorsal striatum (Schatz et al. 2000). Direct evidence showing that NOS inhibitors modify c‐Fos‐IR within the striatum following an acute catalepsy effect has not yet been reported. However, a previous study with the neuronal NOS (nNOS) inhibitor 7‐nitroindazole (7‐NI) showed attenuated c‐Fos‐IR in the striatum and NAc following forced swimming stress (Silva et al. 2012). Our group has previously reported that the cross‐tolerance observed between the nonselective NOS inhibitor, L‐NOARG (NG‐nitro‐L‐arginine), and clozapine, an atypical antipsychotic, was accompanied by decreased FosB/ΔFosB protein immunoreactivity in the dorsal striatum and NAc shell region, along with reduced NADPH‐d activity in the dorsal and ventral lateral striatum (VL) (Prieto et al. 2019). Furthermore, 7‐NI has been reported to prevent L‐DOPA‐induced FosB/ΔFosB upregulation in NADPH‐d‐positive interneurons in the DL and DM striatum (Padovan‐Neto et al. 2015), suggesting, to some extent, a colocalization of NADPH‐d/nNOS‐positive interneurons with FosB/ΔFosB that can be reduced following chronic treatment with a NOS inhibitor in this type of movement disorder.

Based on the following considerations: (1) the dopaminergic modulatory role of NO in the striatum; (2) evidence that NOS inhibitors can mimic the catalepsy induced by D2R antagonism; (3) the limited number of studies examining striatal c‐Fos‐IR distribution with MCP and the absence of such studies with L‐NOARG; and (4) the lack of studies addressing NOS/NADPH‐d activity in parallel with c‐Fos‐IR in catalepsy test following an acute dose, the present study was designed to evaluate motor side effects resulting from the acute administration of Hal, MCP, and L‐NOARG at different, well‐established preclinical doses. Furthermore, we investigated whether these motor side effects could be correlated with changes in the number of c‐Fos‐IR and nNOS‐positive cells, as well as NADPH‐d activity in the striatum and NAc. We hypothesize that the emergence of cataleptic behaviors following these treatments is associated with region‐specific changes in c‐Fos‐IR and NOS/NADPH‐d activity across striatal subregions.

## 2 Materials and Methods

### 2.1 Animals

Sixty‐five male Swiss mice (30–35 g) were housed in groups of two or three per cage, undergoing environmental enrichment and quarantine for a period of 7 days. They were kept in a temperature‐ and humidity‐controlled room with a 12‐h light/dark cycle (lights on at 7:00 a.m.) and with unlimited access to water and food. Handling of all animals and experiment protocols was in accordance with the guidelines outlined in the Ethics Committee on Animal Use (CEUA‐UFABC) protocol No. 1362060218. All efforts were made to minimize animal suffering. Experiments were performed between 9:00 a.m. and 6 p.m. They were conducted in a soundproof, temperature‐controlled room, illuminated with two 40 W fluorescent lights placed 1.3 m from the animals. The experimental apparatus was cleaned and dried before each session using a 10% ethanol solution to minimize olfactory cues. Each animal underwent the full experimental pipeline and contributed data to both behavioral and histological analyses. The sample size was determined based on previous studies with a similar experimental design (Prieto et al. 2019). Moreover, all observed effect sizes were consistent with a statistical power of 80% at a significance level of 0.05. The free G*Power software was used to calculate effect sizes.

### 2.2 Drug Administration

For this study, mice were randomly allocated to treatment groups using alternation. Randomization was also applied to determine the order of animals during behavioral testing to minimize potential order effects, and the animals were then assigned to three different groups: Hal (Haldol, Janssen), MCP (Plasil Sanofi Adventis), both D2‐like receptor antagonists; and L‐NOARG (Sigma‐Aldrich, St. Louis, Missouri, USA), a NOS inhibitor. All drugs were administered via intraperitoneal (ip) injection at a volume of 10 mL/kg, with saline (Sal) serving as vehicle and control. Different doses were given to each experimental group, as described below and in Table 1.

- Experiment 1: Sal or Hal at 0.1, 0.5, or 1 mg/kg (*n* = 5 per dose), referred to as Hal0.1, Hal0.5, and Hal1 (Banasikowski and Beninger 2012; Hiroi and Graybiel 1996; Nguyen et al. 1992; Starr and Starr 1987).
- Experiment 2: Sal or MCP at 1, 5, 8, or 45 mg/kg (*n* = 5 per dose), referred to as MCP1, MCP5, MCP8, and MCP45 (Ahmad Khan et al. 2004; Baker et al. 2014; Hiroi and Graybiel 1996).
- Experiment 3: Sal or L‐NOARG at 15, 30, or 45 mg/kg (*n* = 5 per dose), referred to as LN15, LN30, and LN45 (De Oliveira et al. 1996; Prieto et al. 2019).

**TABLE 1:** Summary of the treatment schedules.

Drug	Dose	*N*	Behavior test	Histological test
Saline	10 mL/kg/ip	15 (5 per each treatment)	Catalepsy Rota‐rod	c‐Fos‐IR nNOS‐IR NADPH‐diaphorase
Haloperidol	0.1, 0.5, and 1 mg/kg/ip	5 per dose		
Metoclopramide	1, 5, 8, and 45 mg/kg/ip	5 per dose		
L‐NOARG	15, 30, and 45 mg/kg/ip	5 per dose		

After treatments were administered, catalepsy was assessed at 5‐, 30‐, 60‐, 90‐, and 120‐min postinjection, and Rota‐rod performance was assessed at 65‐min postinjection. Following the last catalepsy test, animals were euthanized and perfused for brain extraction as described below. In our pilot experiments, doses of L‐NOARG up to 45 mg/kg did not increase mortality rates. For MCP, a dose of 8 mg/kg in mice corresponds to approximately 40 mg/day in humans (Friedman et al. 2011), according to the translation formula of Reagan‐Shaw et al. (2007).

### 2.3 Behavioral Procedures

#### 2.3.1 Catalepsy Test

Each animal was tested by placing both forelimbs on a horizontal cylindrical glass bar (diameter: 0.5 cm; height: 4.5 cm above the table) (Echeverry et al. 2007; Prieto et al. 2019; Sanberg et al. 1988). The latency (in seconds—s) during which both forelimbs remained on the bar was recorded, up to a maximum of 300 s (Zarrindast et al. 1993). Catalepsy was considered to have ended when at least one forepaw touched the floor or when the mouse climbed onto the bar.

#### 2.3.2 Rota‐Rod Test

Motor coordination and balance were evaluated using a Rota‐rod (Panlab model LE8500). The test involved placing each mouse on a rotating drum and measuring the time it maintained balance while walking on the rod. Animals were pretrained for 3 days prior to the test. An animal was included only if it had already completed the maximum training period of 300 s; therefore, normalization to baseline was not necessary. The rotarod rotated at a speed of 8 rpm for a maximum of 300 s. During each session, animals were gently touched on their tails several times to maintain a high level of alertness (Dekundy et al. 2007; Prieto et al. 2019). Behavioral assessments were conducted without experimenter blinding.

### 2.4 Tissue Preparation

Mice were anesthetized with 30% urethane (Sigma‐Aldrich, St. Louis, MO, USA) and transcardially perfused with 100 mL of 0.01 M PBS, followed by 100 mL of 4% paraformaldehyde (Sigma‐Aldrich, St. Louis, MO, USA) in 0.1 M phosphate buffer (PB) (pH 7.4). Brains were rapidly removed, postfixed in 4% paraformaldehyde for 2 h, and then cryoprotected in 30% sucrose/PB for 48 h at 4°C. Brains were quickly frozen in cold isopentane (−40°C; Sigma‐Aldrich, St. Louis, MO, USA) and stored at −80°C until further processing. Serial coronal sections 30 μm thick were cut on a cryostat (CM1850 Leica). Sections through the striatum were collected in ethylene glycol antifreezing solution and stored at −20°C until use (Prieto et al. 2019). c‐Fos, nNOS immunostaining, and NADPH‐d activity were performed on different but adjacent brain sections containing the caudate–putamen complex (CPu or striatum) and NAc along the anterior–posterior (AP) axis, as described below, for all animals used in the behavioral tasks.

### 2.5 Immunohistochemistry

It was performed using a standard peroxidase‐based method (Prieto et al. 2019). To unmask antigens and epitopes, a citrate buffer antigen retrieval protocol was used. Sections were incubated overnight at 4°C with either c‐Fos rabbit antibody (1:1000 sc‐253, Santa Cruz Biotechnology) or neuronal nitric oxide synthase (nNOS) rabbit antibody (1:500, sc‐648, Santa Cruz Biotechnology), followed by incubation with biotinylated goat antirabbit IgG secondary antibody (BA‐5000‐1.5, Vector Laboratories, Southfield, MI, USA) and HRP‐conjugated streptavidin (Vectastain Kit Elite ABC peroxidase, PK 6100, Vector Laboratories, Southfield, MI, USA). Sections were developed using 3,3′‐diaminobenzidine (D12384‐25G, DAB; Sigma, 0.5 mg/mL) and 0.03% hydrogen peroxide. Tissues from all experiments were processed in the same assay. Slices were mounted on slides and cover‐slipped for microscopic analysis.

### 2.6 NADPH‐d Histochemistry

NADPH‐d histochemical activity (nicotinamide adenine dinucleotide phosphate‐diaphorase activity of nNOS) was analyzed using the method described by Echeverry et al. (2004). Briefly, free‐floating slices were preincubated in 0.1 M PB for 10 min. Sections were then incubated in an NADPH‐d solution containing 0.7‐mg/mL b‐NADPH (Calbioquem), 50‐mg/mL nitroblue tetrazolium (Sigma‐Aldrich), and 0.2 M PB with 3% TritonX‐100, continuously shaken at 37°C for 2 h. The reaction was stopped by the addition of 0.1 M PB.

### 2.7 Quantification

A preliminary qualitative analysis of all brain sections was performed to identify bilateral labeling and brain plates. Structure localization was determined according to Paxinos and Franklin (2008). Images with positive immunostaining for c‐Fos and nNOS (visualized via a dark brown reaction product) or NADPH‐d‐positive staining (visualized via a dark blue reaction product) were digitally captured with a Leica DFC295 camera at 10× magnification connected to a Leica 5500 DMB microscope. Image analysis was performed using a computerized system (open‐source image processing program, ImageJ—NIH System—https://imagej.net/ij/download.html). The striatum was divided into three sections for analysis: dorsolateral (DL), dorsomedial (DM), and ventrolateral (VL) sections (Figure 2F) (Burton et al. 2015). The NAc was subdivided into core and shell regions (Figure 2F) (Shilliam and Dawson 2005). For c‐Fos and nNOS staining and NADPH‐d activity, a minimum of three bilateral sections from each animal were selected for counting positive cells. Selection was based on an optical density threshold of nuclear puncta for c‐Fos and on cell morphology for nNOS staining and NADPH‐d activity, according to the corresponding coordinates along the AP axis (bregma 0.86, 0.74, and 0.62 mm). The experimenter performing the counts was blinded to the treatment group at the time of analysis. The average value from these sections was used for subsequent statistical analyses.

### 2.8 Statistical Analysis

All values are expressed as mean ± standard error of the mean (SEM). Data were tested for unequal variance and normality and log‐transformed (with the addition of a constant value of 1 for catalepsy results) (Echeverry et al. 2007). Catalepsy test data were analyzed via a two‐way repeated‐measures ANOVA, with Time (postinjection) as a within‐subjects factor (five levels) and Treatment as a between‐subjects factor. A one‐way ANOVA was conducted on the Rota‐rod test data for each experimental group. One‐way ANOVA was also used to assess the differences in the number of positive nuclei and cells within specific striatal and NAc regions. The Bonferroni test was applied for multiple comparisons between treatment conditions and the control group. Pearson correlation coefficients were used to examine associations between catalepsy and Rota‐rod performance, as well as between catalepsy at 120 min and cell immunoreactivity or NADPH‐d activity for each striatal region. Additional Pearson correlations were performed to assess the relationship between nNOS immunostaining and NADPH‐d activity. Linear regression was employed following correlation coefficient analysis to evaluate the significance of each predictor and identify that best predicts the behavioral outcome. A *p*‐value below 0.05 was considered statistically significant. OriginPro 8.5 was used for scientific graph generation, and JASP 0.19.3 was used for all statistical analyses.

## 3 Results

### 3.1 Catalepsy Test

The main behavioral ANOVA results are provided in Table S1.

**FIGURE 1:**
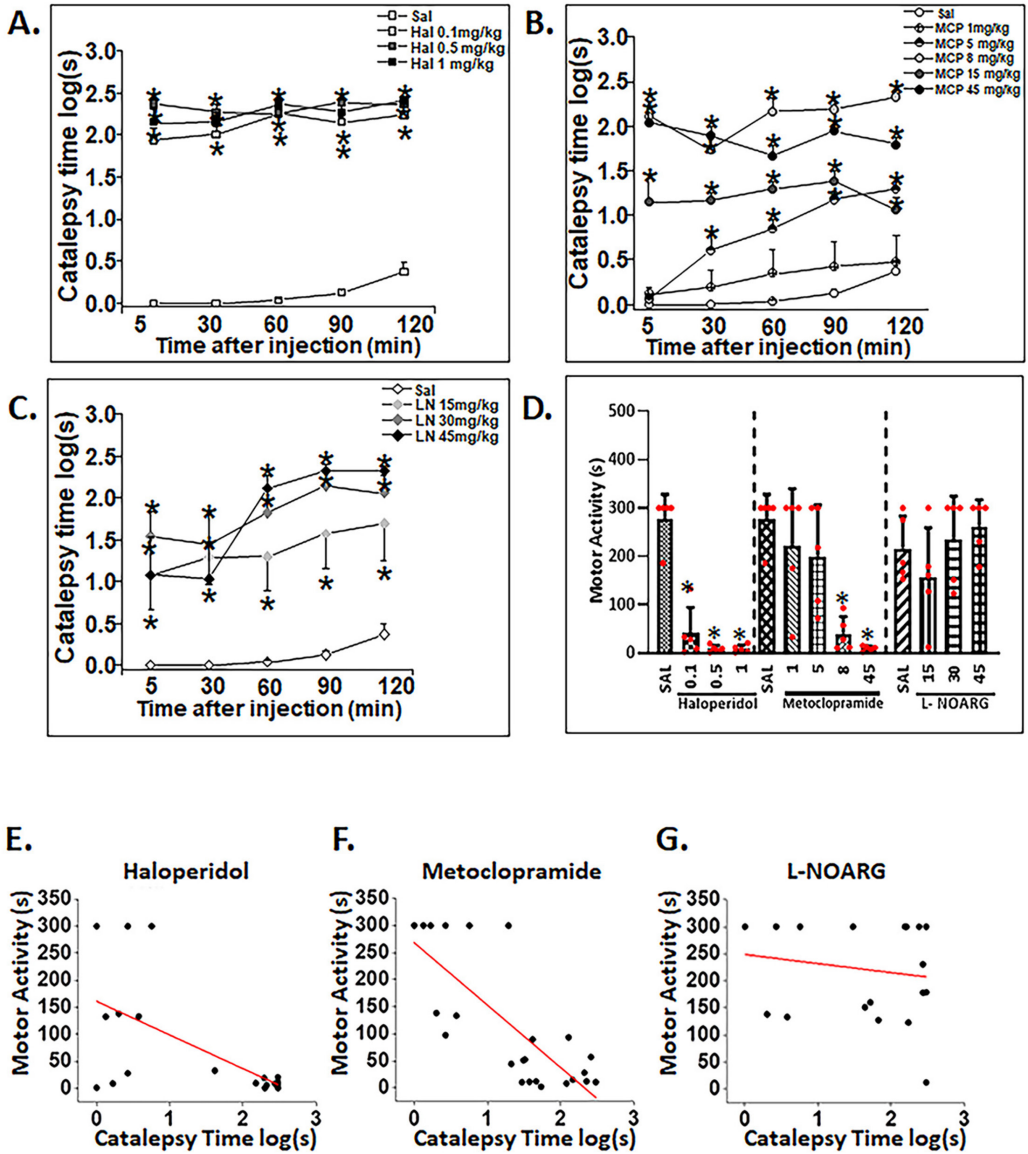
Motor behavior: Catalepsy time (A–C) and time spent on Rota‐rod (D) in mice treated with saline, haloperidol, metoclopramide, or L‐NOARG. Catalepsy time was analyzed by two‐way repeated‐measures ANOVA and Rota‐rod time by one‐way ANOVA for each treatment. For multiple comparisons, the Bonferroni test was applied. All doses of Hal (Panel A), and MCP5, MCP8, and MCP45 (Panel B), and all doses of L‐NOARG (Panel C) induced cataleptic effects compared with the saline group over time. All doses of Hal, as well as MCP8 and MCP45, decreased Rota‐rod performance, whereas L‐NOARG treatment produced no changes (Panel D). A significant negative correlation between catalepsy time and Rota‐rod activity was observed for Hal treatment (Panel E) and MCP (Panel F), but not for L‐NOARG treatment (Panel G) (Pearson correlation, simple linear regression). Treatments were administered intraperitoneally (ip) 5 min before the catalepsy test. *Mean difference compared with control group (Bonferroni: *p* < 0.05). Data are presented as mean ± SEM (*n* = 5 animals/per dose). Hal: haloperidol, LN: L‐NOARG, MCP: metoclopramide, Sal: saline.

The acute administration of Hal, MCP, and L‐NOARG induced catalepsy in a dose‐dependent manner. All doses of Hal produced catalepsy, with significant main effects of Time and Treatment, as well as a significant Time × Treatment interaction (*p* < 0.002). Significant differences were found between all Hal treatments and the Saline (Sal) group, with Hal administration producing a significant increase in catalepsy duration compared with Sal (Bonferroni: *p* < 0.05) (Figure 1A).

Administration of MCP also resulted in a significant increase in catalepsy latency, with significant main effects of Time and Treatment, as well as a significant Time × Treatment interaction (*p* < 0.001). However, Bonferroni post hoc testing indicated no significant effect of the lower dose (MCP 1 mg/kg, MCP1) compared with Sal (Bonferroni: *p* > 0.05) (Figure 1B). Analysis of L‐NOARG (LN) administration also showed significant main effects of Time and Treatment (*p* < 0.001), with all doses significantly increasing catalepsy duration compared with Sal (Bonferroni: *p* < 0.05) (Figure 1C).

### 3.2 Rota‐Rod Test

As shown in Figure 1D, acute administration of Hal (*p* < 0.001) and MCP (*p* < 0.01) decreased the latency to fall from the Rota‐rod, an effect not observed with L‐NOARG administration. All doses of Hal produced significantly lower Rota‐rod latencies compared with Sal (Bonferroni: *p* < 0.05, Figure 1D). However, only intermediate and higher doses of MCP (MCP8 and MCP45, respectively) decreased Rota‐rod latency relative to Sal (Bonferroni: *p* < 0.05, Figure 1D). A significant negative correlation between catalepsy time and Rota‐rod performance was observed following Hal treatment (Pearson *r* = −0.608, *p* < 0.001, Figure 1E), and MCP administration (Pearson *r* = −0.792, *p* < 0.001, Figure 1F), but not after L‐NOARG injection (Pearson *r* = −0.173, *p* = 0.466, Figure 1G). Simple linear regression including both behavioral variables showed that catalepsy time significantly predicted reduced or impaired motor performance on the Rota‐rod, for both Hal (*p* < 0.01) and MCP (*p* < 0.001), but not for L‐NOARG.

### 3.3 c‐Fos‐IR

The main histochemistry ANOVA results are provided in Table S2.

**FIGURE 2:**
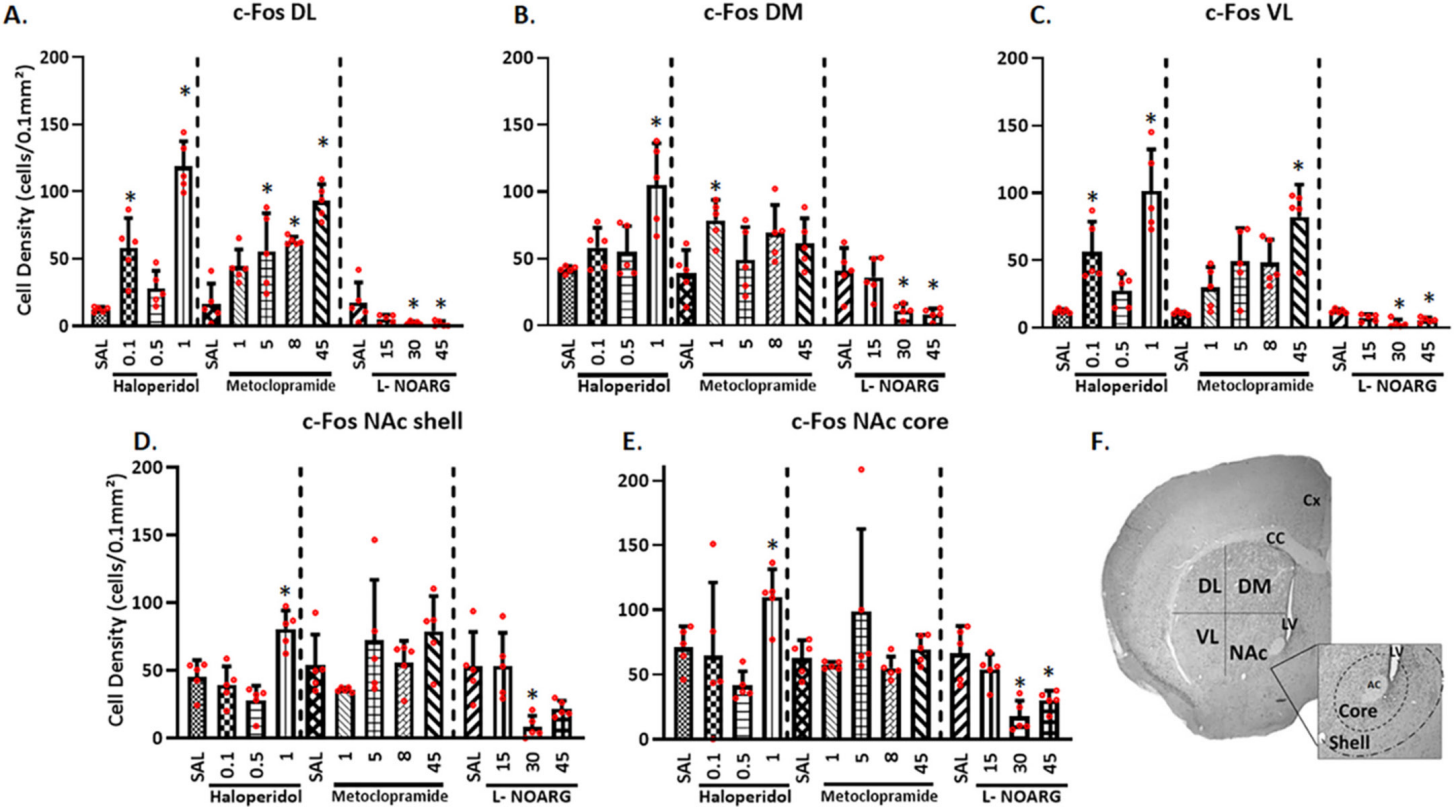
Histological results: c‐Fos‐IR cell density (cells/0.1 mm^2^), in mice treated with saline, haloperidol, MCP, or L‐NOARG. All the cell densities were analyzed by one‐way ANOVA for each treatment. For multiple comparisons, the Bonferroni test was applied. *Mean difference compared with the saline group (Bonferroni: *p* < 0.05). Data are presented as mean ± SEM (*n* = 5 animals/per dose). (F) Schematic representation of the striatal regions analyzed. AC: anterior commissure, CC: corpus callosum, Cx: cortex, DL: dorsolateral, DM: dorsomedial, LV: lateral ventricle, NAc: nucleus accumbens (shell and core regions), Sal: saline; VL: ventrolateral.

**FIGURE 3:**
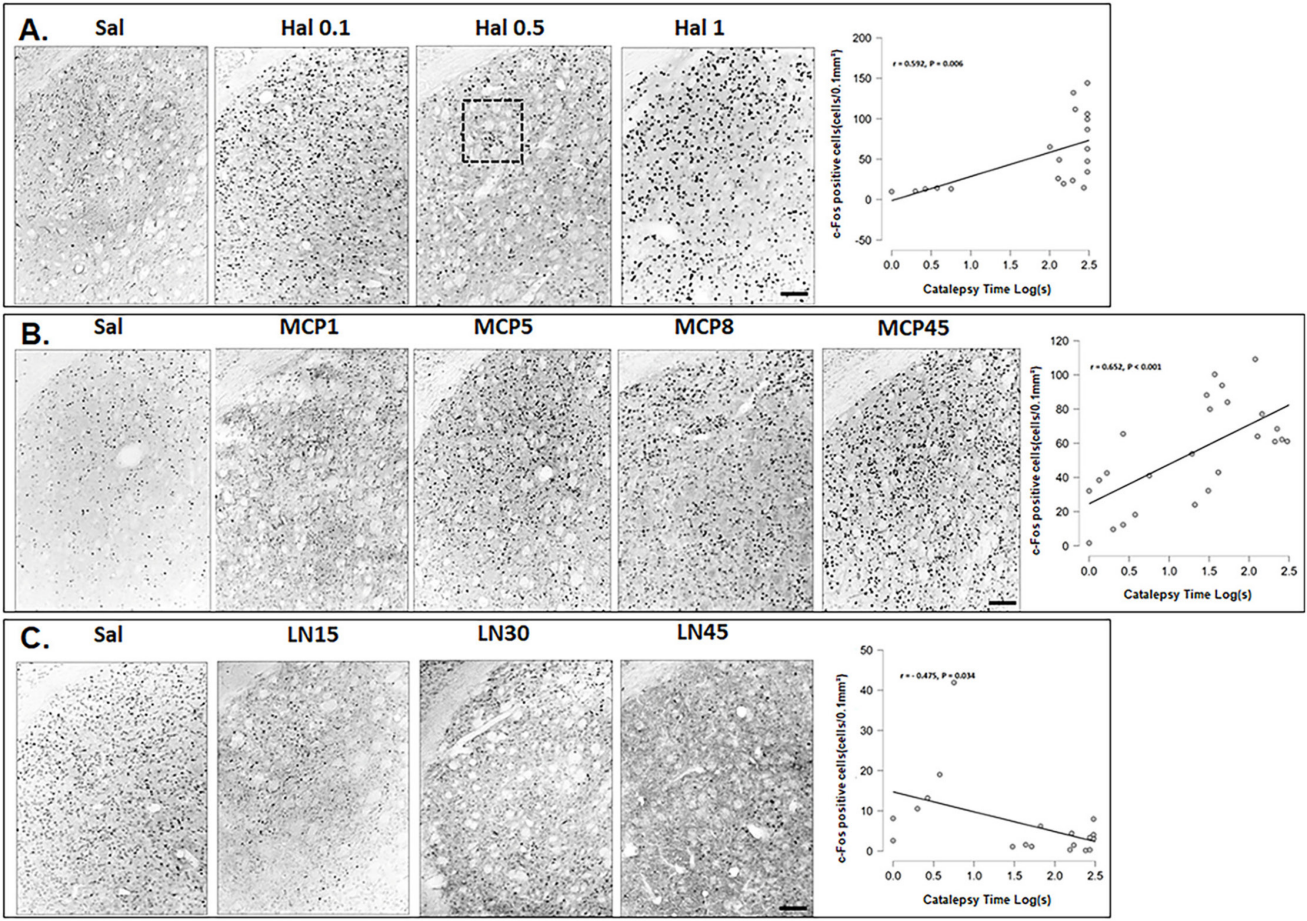
Representative photomicrographs after Saline or Hal (A), saline or MCP (B), and saline or L‐NOARG (C) of c‐Fos immunoreactivity (c‐Fos‐IR) in the dorsolateral striatum, along with correlations between the number of c‐Fos‐positive cells and catalepsy time (Pearson correlation, catalepsy time vs. c‐Fos positive cells in the DL region). Scale bar = 100 μm.

Figure 2 shows the results (mean ± SEM) for c‐Fos‐IR after all treatments. Representative photomicrographs of the DL region analyzed with Pearson correlation coefficients are shown in Figure 3. A significant increase in c‐Fos‐IR was observed in the DL striatum following acute administration of Hal0.1 and Hal1 (*p* < 0.001; Bonferroni: *p* < 0.05, Figure 2A), and after all MCP doses except MCP1 (*p* < 0.001; Bonferroni: *p* < 0.05, Figure 2A). In the DM striatum, c‐Fos‐IR was increased by Hal1 (*p* < 0.001; Bonferroni: *p* < 0.05, Figure 2B) and MCP1 (*p* < 0.05; Bonferroni: *p* < 0.05, Figure 2B). In contrast, L‐NOARG reduced c‐Fos‐IR in the DL striatum at LN30 and LN45 (*p* < 0.05; Bonferroni: *p* < 0.05, Figure 2A), as well as in the DM region (*p* < 0.001; Bonferroni: *p* < 0.05, Figure 2B).

A significant increase in c‐Fos positive cells was observed in the VL striatum following administration of Hal0.1 and Hal1 (*p* < 0.001; Bonferroni: *p* < 0.05, Figure 2C), similar to the DL region, and in shell and core regions of the NAc after Hal1 (shell [*p* < 0.001, Figure 2D]; core [*p* < 0.05; Bonferroni: *p* < 0.05, Figure 2E]), resembling the pattern observed in the DM striatum. The higher dose of MCP, 45 mg/kg, resulted in significant increases in c‐Fos‐IR in the VL striatum (*p* = 0.002; Bonferroni: *p* < 0.05, Figure 2C). In contrast, L‐NOARG at 30 and 45 mg/kg significantly decreased c‐Fos‐IR in the VL striatum (*p* = 0.001; Bonferroni: *p* < 0.05, Figure 2C) and in the core region (*p* < 0.001; Bonferroni: *p* < 0.05, Figure 2E). At 30 mg/kg, L‐NOARG also reduced c‐Fos positive cells in the shell region (*p* = 0.002; Bonferroni: *p* < 0.05, Figure 2D).

The proto‐oncogene c‐Fos is commonly used as an index of neuronal activation in behavioral responses to environmental stimuli (Weinberg et al. 2008). Regional patterns of c*‐*Fos activation were also examined via Pearson correlation analysis (catalepsy time vs. c‐Fos‐positive cells). In the DL region, a positive correlation was observed following acute treatment with Hal (Pearson *r* = 0.592, *p* = 0.006, Figure 3A). Similarly, MCP treatment produced a positive correlation (Pearson *r* = 0.652, *p* < 0.001, Figure 3B). In contrast, L‐NOARG administration was associated with a negative correlation in the DL region (Pearson *r* = −0.475, *p* = 0.034, Figure 3C).

### 3.4 nNOS Positive Cells

**FIGURE 4:**
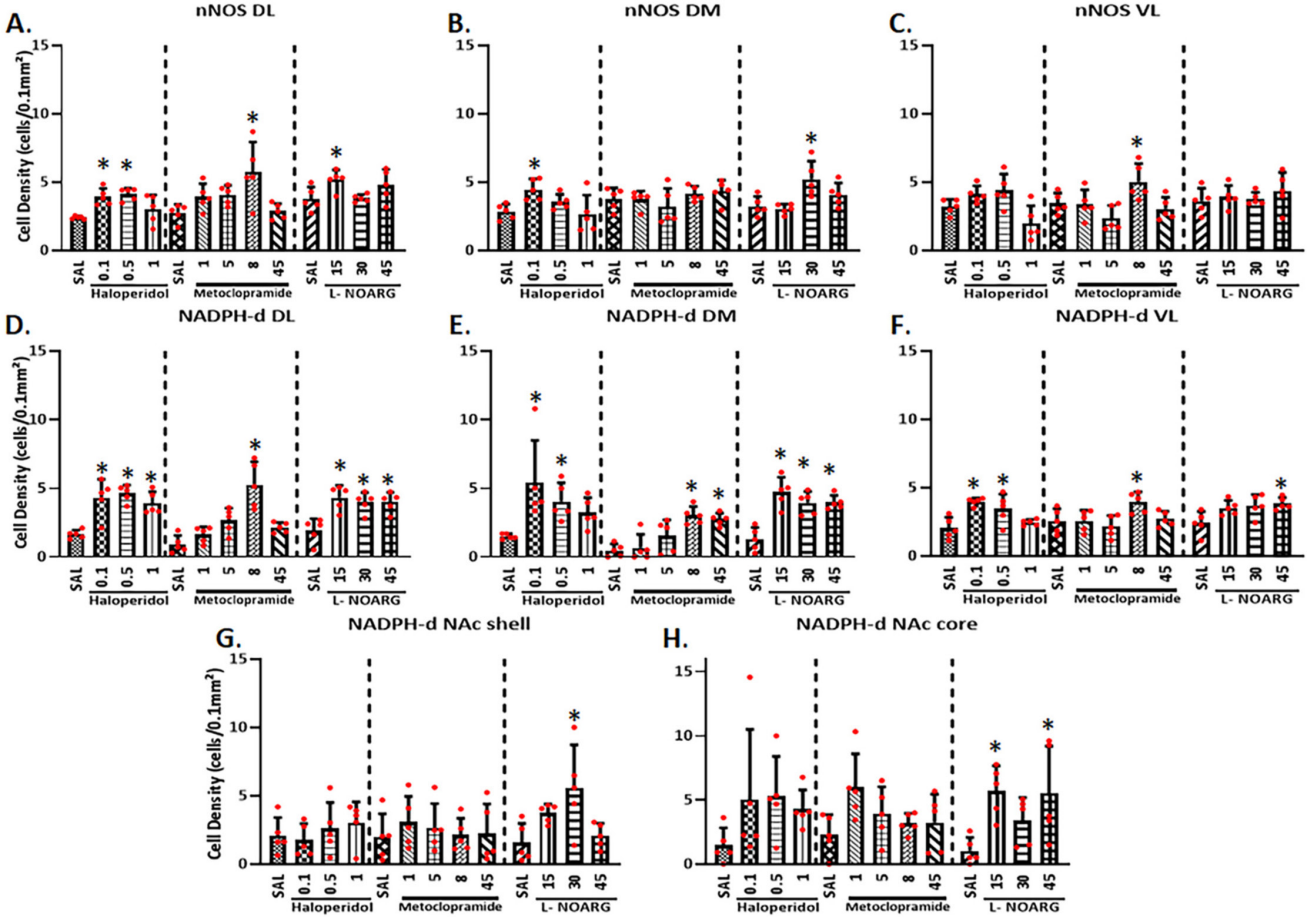
Histological results of nNOS cell density (cells/0.1 mm^2^) (A–C), and NADPH‐d activity (cells/0.1 mm^2^) (D–H), in mice treated with saline, haloperidol, MCP, or L‐NOARG. All cell densities were analyzed by one‐way ANOVA for each treatment. For multiple comparisons, the Bonferroni test was applied. *Mean difference compared with the saline group (Bonferroni: *p* < 0.05). Data are presented as mean ± SEM (*n* = 5 animals/per dose). Sal: saline.

**FIGURE 5:**
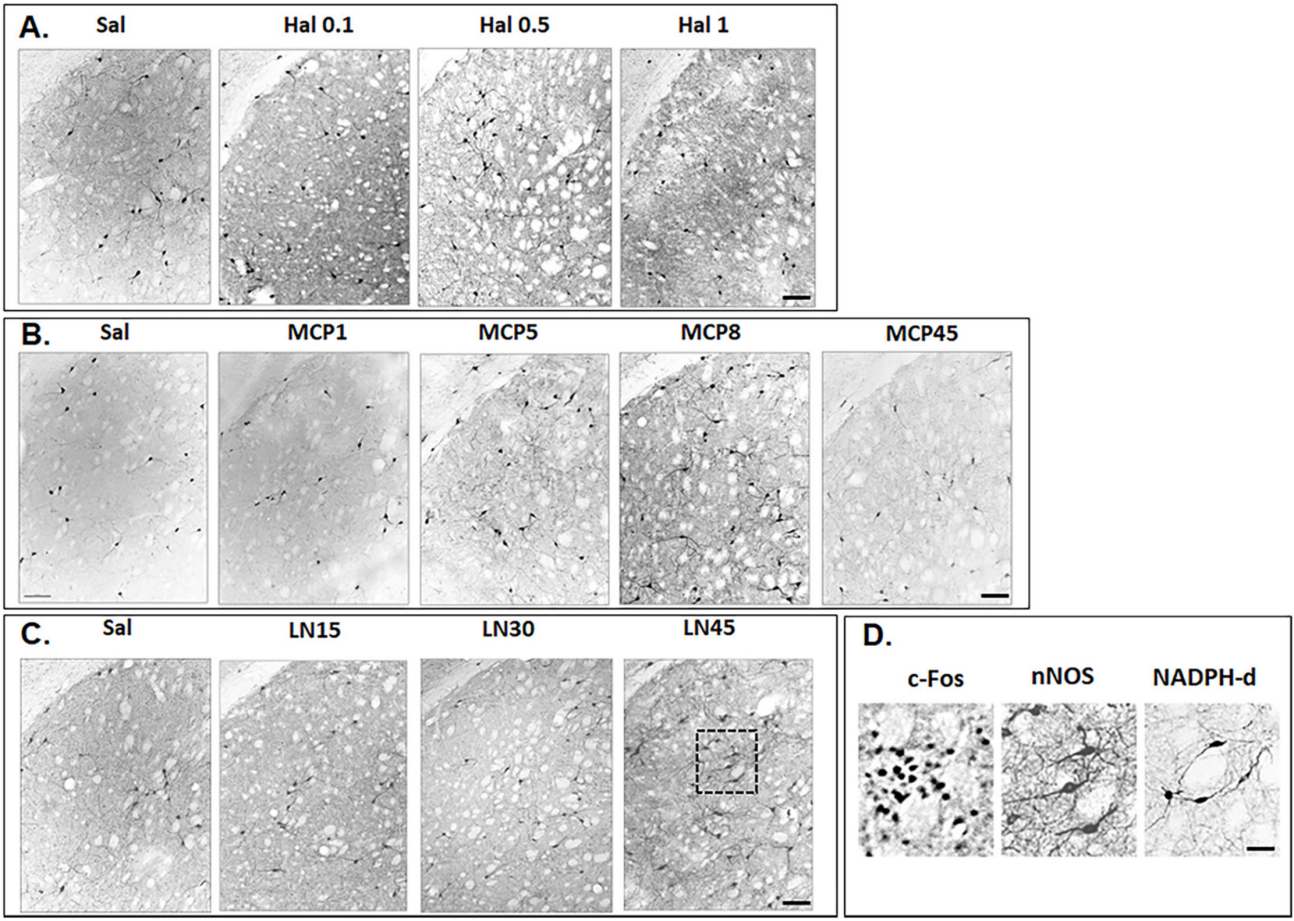
Representative photomicrographs after saline or Hal (A), saline or MCP (B), and saline or L‐NOARG (C) of nNOS immunoreactivity in the dorsal lateral striatum. Scale bar = 100 μm. Panel D: Striatal staining for c‐Fos, nNOS, and NADPH‐d activity. Scale bar = 50 μm.

Figure 4A–C shows the number of nNOS‐positive cells (mean ± SEM) after each treatment. Representative photomicrographs of the DL region are shown in Figure 5. In the DL striatum, acute administration of Hal0.1 and Hal0.5 (*p* = 0.002; Bonferroni *p* < 0.05, Figure 4A), MCP8 (*p* = 0.005; Bonferroni *p* < 0.05), and LN15 (*p* = 0.037; Bonferroni: *p* < 0.05, Figure 4A) significantly increased nNOS labeling. In the DM region, increased numbers of nNOS‐positive cells were observed after Hal0.1 (*p* = 0.021; Bonferroni *p* < 0.05, Figure 4B), and LN30 treatment (*p* = 0.005; Bonferroni: *p* < 0.05, Figure 4B), whereas MCP produced no significant changes. In the VL region, MCP8 significantly increased nNOS staining (*p* = 0.007; Bonferroni *p* < 0.05, Figure 4C), whereas neither Hal nor L‐NOARG altered nNOS labeling (Bonferroni *p* > 0.05). No significant changes were detected in the core and shell regions of the NAc (*p* > 0.05).

### 3.5 NADPH‐d Activity

**FIGURE 6:**
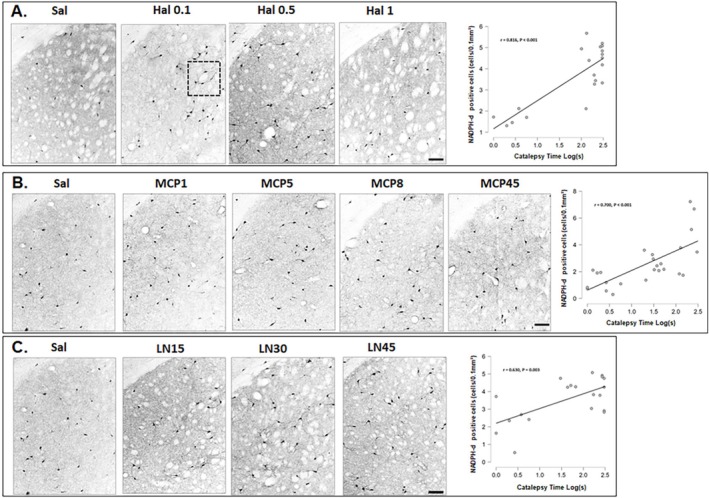
Representative photomicrographs after saline or Hal (A), saline or MCP (B), and saline or L‐NOARG (C) for NADPH‐diaphorase activity cells in the dorsolateral striatum, along with correlations between the number of NADPH‐d activity cells and catalepsy time (Pearson correlation, catalepsy time vs. NADPH‐d activity cells in the DL region). Scale bar = 100 μm.

Figure 4D–H shows the mean ± SEM for the number of NADPH‐d activity cells following each treatment used in the present study. Representative photomicrographs of the DL region analyzed with Pearson correlation coefficients are shown in Figure 6. NADPH‐d activity significantly increased in the DL striatum after all doses of Hal (*p* < 0.001; Bonferroni: *p* < 0.05, Figure 4D) and in the DM region at Hal0.1 and Hal0.5 (*p* < 0.001; Bonferroni: *p* < 0.05, Figure 4E). Increased NADPH‐d activity was observed in the DL region following MCP8 treatment (*p* < 0.001; Bonferroni: *p* < 0.05, Figure 4D) and in the DM region at MCP8 and MCP45 (*p* < 0.001; Bonferroni: *p* < 0.05, Figure 4E). All doses of L‐NOARG significantly increased NADPH‐d activity in both the DL (*p* < 0.001; Bonferroni: *p* < 0.05, Figure 4D) and DM (*p* < 0.001; Bonferroni: *p* < 0.05, Figure 4E) regions. In VL striatum, NADPH‐d activity was enhanced after Hal0.1 and Hal0.5 (*p* = 0.001; Bonferroni: *p* < 0.05, Figure 4F), MCP8 (*p* = 0.017; Bonferroni: *p* < 0.05, Figure 4F), and LN45 (*p* = 0.018; Bonferroni: *p* < 0.05, Figure 4F). In the NAc shell region, significant increases were observed only for LN30 (*p* = 0.012; Bonferroni: *p* < 0.05, Figure 4G), whereas in the NAc core region, significant increases were observed only for LN15 and LN45 (*p* = 0.020; Bonferroni: *p* < 0.05, Figure 4H).

**FIGURE 7:**
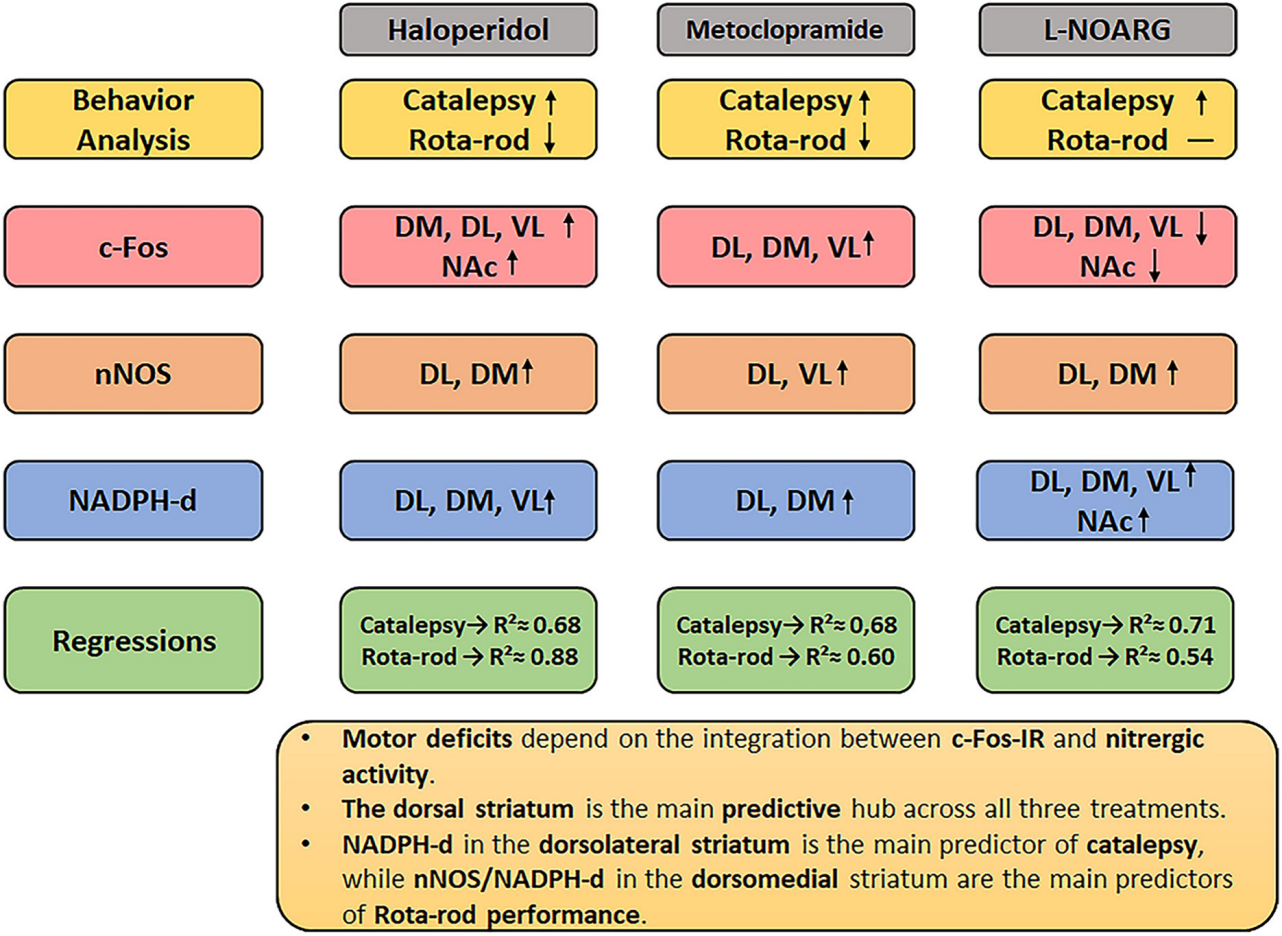
Summary of the effects of different doses of Hal, MCP, or L‐NOARG on the induction of c‐Fos‐IR, nNOS, and NADPH‐d activity in striatal and nucleus accumbens (NAc) regions. Regression results are also summarized.

Pearson correlations between catalepsy time and NADPH‐d activity revealed a positive correlation in the DL region following Hal administration (*r* = 0.816, *p* < 0.001), as well as after MCP (*r* = 0.700, *p* < 0.001) and L‐NOARG (*r* = 0.630, *p* = 0.003) administration (Figure 6A–C). Additionally, correlations between nNOS and NADPH‐d were performed to determine in which regions the increase of nNOS was associated with increased NADPH‐d activity. In the DL region, a positive correlation was observed for HAL (*r* = 0.577, *p* < 0.01) and MCP (*r* = 0.700, *p* < 0.001), whereas in the NAc shell region, a positive correlation was found for L‐NOARG (*r* = 0.487, *p* < 0.05). Figure 7 summarizes the results of c‐Fos, nNOS, and NADPH‐d activity for all groups.

### 3.6 Linear Regressions

A multivariable regression analysis was performed after collecting all the variables within each comparison group. In this part, we aimed to determine which regions, proteins, and activity levels were the best predictors of catalepsy or Rota‐rod performance. The best‐fitting models were generated separately for each treatment.

#### 3.6.1 Hal

A multiple linear regression was conducted to predict catalepsy at 120 min using c‐Fos‐IR and NADPH‐d in the DL as predictors. The overall model was statistically significant, *F*(2, 17) = 18.13, *p* < 0.001, explaining 68.1% of the variance in the outcome (*R* ^2^ = 0.681; adjusted *R* ^2^ = 0.643) (Table 2). Collinearity diagnostics indicated that both predictors had tolerance values ≈ 0.838 and VIF ≈ 1.19, which are well within acceptable limits, suggesting no multicollinearity concerns.

**TABLE 2:** Summary of the linear regression coefficients within the groups.

Predictor	*B*	SE	*T*	*p*
Hal—Catalepsy 120 min				
Intercept	−66.44	45.38	−1.46	0.161
c‐Fos DL	1.011	0.411	2.461	0.025
NADPH‐d DL	51.226	12.676	4.041	< 0.001
Hal—Rota‐rod				
Intercept	234.5	65.22	3.6	0.003
c‐Fos DM	−2.05	0.4	−5.12	< 0.001
c‐Fos NAc core region	1.25	0.37	3.36	0.005
nNOS DL	−72.40	14.33	−5.05	< 0.001
nNOS NAc shell	49.21	9.34	5.27	< 0.001
MCP—Catalepsy 120				
Intercept	−104.22,	31.895	−3.27	0.004
nNOS VL	26.2	9.099	2.88	0.009
NADPH‐d DL	32.31	6.664	4.85	< 0.001
MCP—Rota‐rod				
Intercept	273.9	60.278	4.545	< 0.001
c‐Fos DM	−2.347	0.803	−2.922	0.008
nNOS NAc core region	21.105	8.586	2.458	0.023
NADPH‐d DM	−47.206	14.08	3.353	0.003
L‐NOARG—Catalepsy 120 min				
Intercept	−410.52	144.68	−2.84	0.012
c‐Fos DM	−3.937	1.11	−3.55	0.003
c‐Fos NAc shell region	2.327	0.956	2.43	0.028
nNOS DL	57.034	18.102	3.15	0.007
nNOS DM	76.819	19.857	3.87	0.002
L‐NOARG—Rota‐rod				
Intercept	396.49	44.57	8.9	< 0.001
NADPH‐d DM	−26.07	9.94	−2.62	0.018
c‐Fos NAc shell region	−2.31	0.58	−3.96	0.001

A second multiple linear regression was performed to predict Rota‐rod performance using c‐Fos‐IR in the DM and NAc core, nNOS in the DL and NAc shell, and NADPH‐d activity in the NAc core regions as predictors. The model was also statistically significant, *F*(5, 14) = 19.89, *p* < 0.001, accounting for 87.7% of the variance (*R* ^2^ = 0.877; adjusted *R* ^2^ = 0.832) (Table 2). Multicollinearity diagnostics showed all tolerance values above 0.68 and VIF values between 1.09 and 1.14, again indicating no multicollinearity concern.

#### 3.6.2 MCP

A multiple linear regression was conducted to predict catalepsy at 120 min from nNOS in the VL and NADPH‐d activity in the DL. The overall model was statistically significant, *F*(2, 22) = 23.59, *p* < 0.001, accounting for 68.2% of the variance in the outcome (*R* ^2^ = 0.682; adjusted *R* ^2^ = 0.653) (Table 2). Multicollinearity diagnostics indicated no concerns, with both predictors showing tolerance ≈ 0.88 and VIF ≈ 1.14. A second multiple linear regression was conducted to predict Rota‐rod performance from c‐Fos in the DM, nNOS in the NAc core region, and NADPH‐d in the DM. This model was also significant, *F*(3, 24) = 10.66, *p* < 0.001, explaining 60.4% of the variance in the outcome (*R* ^2^ = 0.604; adjusted *R* ^2^ = 0.547) (Table 2). Multicollinearity diagnostics again indicated no concerns, with all predictors showing tolerance ≈ 0.89 and VIF ≈ 1.11.

#### 3.6.3 L‐NOARG

A multiple linear regression analysis was performed to predict catalepsy at 120 min from c‐Fos‐IR in the DM and NAc shell region, nNOS in the DL, and DM. The overall model was statistically significant, *F*(4, 15) = 9.28, *p* < 0.001, accounted for approximately 71.2% of the variance in the dependent variable (*R* ^2^ = 0.712; adjusted *R* ^2^ = 0.636) (Table 2). Collinearity diagnostics revealed tolerance values ranging from 0.435 (c‐Fos in the NAc shell region) to 0.873 (nNOS in the DL), while corresponding VIF values between 1.15 and 2.30, indicating that multicollinearity was not a concern in this model. A second multiple linear regression analysis was conducted to predict Rota‐rod performance using NADPH‐d activity in the DM and c‐Fos‐IR in the NAc shell region as predictors. The overall model was statistically significant, *F*(2, 17) = 10.17, *p* = 0.001, explaining 54.5% of the variance in the outcome (*R* ^2^ = 0.545; adjusted *R* ^2^ = 0.491) (Table 2). Multicollinearity diagnostics showed tolerance values of ≈ 0.986 and corresponding VIF values of ≈ 1.02, again indicating no concerns with multicollinearity.

## 4 Discussion

### 4.1 Behavioral Outcomes

In the present study, we showed that mild doses of MCP, similar to clinically relevant doses, can induce catalepsy and motor balance deficits in a similar way to higher doses of MCP and to Hal, whose cataleptic effects have been linked to striatal D2R occupancy (Wadenberg et al. 2001). Interestingly, L‐NOARG treatment also induced catalepsy but did not affect motor coordination on the Rota‐rod test.

Hal and MCP are used in screening tests to evaluate the clinical potency of neuroleptic drugs and their ability to induce EPS (Iwanami et al. 1981). Although earlier studies reported a cataleptic effect with MCP doses similar to those used here (Ahtee 1975a, 1975b; Ahtee and Buncombe 1974; Casteels‐Van Daele et al. 1970), subsequent research did not observe catalepsy after doses up to 10 mg/kg of MCP. However, both chronic and acute treatment with MCP caused sensitization to catalepsy induced by Hal (Agovic et al. 2008; Imamura et al. 1988), and MCP has not been reported to produce motor behavior alterations (Starr and Starr 1987).

Catalepsy induced by L‐NOARG has been described in studies investigating the interactions between the NO system and Hal. These studies also examined other neurotransmitter systems; for example, studies of side effects and cross interactions (Del‐Bel and Guimarães 2000; Marras et al. 1995; Nucci‐Da‐Silva et al. 1999). The pharmacological interactions between L‐NOARG and potential treatments for dyskinesia in Parkinson's disease (Padovan‐Neto et al. 2009) and schizophrenia (Gupta and Basu 2001; Issy et al. 2011; Wiley 1998) have also been investigated. Low doses of L‐NOARG have been reported to decrease exploration in the open field arena (E. Del Bel et al. 2002) but not induce EPS (Prieto et al. 2019; Wadenberg et al. 2001), whereas higher doses have been associated with reduced exploration in the plus maze (De Oliveira et al. 1996). In contrast, in the present study, mice administered L‐NOARG showed no deficits in motor balance on the Rota‐rod test, similar to effects observed with several NOS inhibitors (Spolidório et al. 2007) and the specific nNOS inhibitor 7‐NI (Padovan‐Neto et al. 2009). Discrepancies between studies using NOS inhibitors may be explained by differences in susceptibility between rats and mice, as well as between strains, similar to observations with dopaminergic drugs (Romanova et al. 2016; Starr and Starr 1987). The motor deficits observed with Hal and higher doses of MCP resemble common side effects of hypoactivity and sedative effects with dopaminergic agents (Joshi et al. 2017; Starr and Starr 1987).

### 4.2 Striatal Activation Patterns (c‐Fos)

There is evidence that the pathophysiology of EPS involves downstream molecules in MSNs (De Bartolomeis et al. 2017; Bateup et al. 2008; Bonito‐Oliva et al. 2011), and elevated c‐Fos‐IR in the DL striatum has been associated with frequently reported motor disabilities induced by typical antipsychotics such as Hal (Deutch et al. 1992; Robertson et al. 1992), including at the doses used here (Rogue and Vincendon 1992). In fact, MCP appears to produce c‐Fos‐IR changes similar to those induced by Hal, with an increased immunolabeling in the DL striatum following cataleptic doses of both drugs. Additionally, c‐Fos‐IR increased in the DM region after higher doses of Hal, as well as after low and intermediate doses of MCP. A positive Pearson correlation between c‐Fos‐IR and catalepsy in the DL striatum was confirmed for both MCP and Hal. While MCP induced c‐Fos in the DM striatum at lower doses, establishing a direct comparison with atypical antipsychotic engagement of this region is challenging due to differences in receptor pharmacology and circuit effects. Previous studies have described the lowest effective dose of Hal as the cataleptic threshold dose (Starr and Starr 1987), and doses in the range of 0.1–0.5 mg/kg have already been used in catalepsy sensitization paradigms (Banasikowski and Beninger 2012). The distribution of c‐Fos‐IR in the dorsal and ventral striatum, as well as in the NAc, following Hal administration has been reported previously (Pinna et al. 1999). In the present study, we confirmed that Hal increases c‐Fos IR in the DL, DM, and VL striatum, but only at the highest dose (1 mg/kg) did we observe a significant increase in c‐Fos labeling in the NAc. MCP treatment resulted in a similar pattern of c‐Fos labeling, with evident staining in DL, DM, and VL regions, but without significant immunoreactivity in the NAc.

In contrast, the higher‐cataleptic doses of L‐NOARG elicited a significant reduction in c‐Fos‐IR in the DL, DM, and VL striatum, as well as in the NAc—an effect opposite to that reported for both D2R blockers in the striatum and for Hal in the NAc. Thus, the negative Pearson correlation observed here between c‐Fos‐IR and catalepsy in DL striatum after L‐NOARG suggests that the relationship among catalepsy, c‐Fos‐IR, and antagonism of striatal D2Rs, as previously described in the literature, may not be applicable to L‐NOARG‐induced catalepsy at the doses used here. Decreased c‐Fos‐IR in dorsal striatum following NOS inhibition has also been reported in a contingency management paradigm with methylphenidate and 7‐NI (Issy and Del Bel 2014). A similar reduction was observed after subchronic administration of L‐NOARG and clozapine when examining striatal FosB/ΔFosB immunoreactivity (Prieto et al. 2019). Both studies, including the contingency paradigm, also showed decreased c‐Fos family immunoreactivity in the NAc shell and core regions, consistent with the findings of the present study in response to higher doses of L‐NOARG.

Taken together, these results suggest that NOS inhibitors modulate neuronal activity in discrete motor‐limbic regions, with the NAc acting as an interface hub, through regulation of Fos family proteins such as c‐Fos and FosB (Tulchinsky 2000). This mechanism of action may occur in response to a single NOS inhibitor or be facilitated by a combination with other neurological and psychiatric drugs (Issy and Del Bel 2014; Padovan‐Neto et al. 2009; Popeski and Woodside 2001; Prieto et al. 2019). Despite our study's limitations, as colocalization was not performed, L‐NOARG appears to reduce c‐Fos labeling in NADPH‐d/nNOS‐positive interneurons, consistent with its reported antidyskinetic effects in L‐DOPA–induced dyskinesia (Padovan‐Neto et al. 2015). This reduction contrasts with the c‐Fos induction observed with Hal and MCP and may reflect recruitment of distinct neuronal populations—such as inhibition of nNOS interneurons by NO blockade versus activation of D2R‐expressing MSNs by D2R antagonists. Although speculative, this framework provides a possible mechanistic explanation for the divergent c‐Fos patterns observed across treatments.

### 4.3 nNOS/NADPH‐d Findings

In the present study, we observed an increase in nNOS immunostaining in the DL striatum after the lowest and intermediate doses of Hal (0.1 and 0.5 mg/kg), the intermediate dose of MCP (8 mg/kg), and the lowest dose of L‐NOARG (15 mg/kg). D2R antagonists have been shown to increase NOS activity (Morris et al. 1997), facilitate NO efflux in the striatum (Sammut et al. 2007), and inhibit NO formation in vitro (Hu et al. 1994). Chronic use of typical antipsychotics suppresses both NO and citrulline levels in the striatum (Bishnoi et al. 2009), and the mechanism of Hal‐induced NOS suppression has been attributed to direct inhibition of NOS enzyme activity (Bishnoi et al. 2009; Nel and Harvey 2003; Hu et al. 1994; J. Zhang et al. 2012). Under such conditions, excessive inhibition of NOS activity, with the resultant decrease in NO levels, can potentiate NMDA receptor‐mediated excitotoxicity (Bishnoi et al. 2009; Connop et al. 1995; West and Galloway 1997), with consistent calcium influx leading to nNOS activation via NMDA receptor signaling (Brenman et al. 1996). MCP‐induced catalepsy has also been associated with D2 and NMDA receptor upregulation in the frontal cortex and striatum (Agovic et al. 2008). In addition, pretreatment with several noncompetitive NMDA antagonists has been shown to inhibit Hal‐induced catalepsy and attenuate the induction of c‐Fos‐IR in the DL, DM, and VL striatal regions (Yanahashi et al. 2004). In the present study, however, acute treatment with Hal and MCP did not reduce the number of nNOS‐positive cells.

Furthermore, NADPH‐d correlates with nNOS activity (Dawson et al. 1991; Hope et al. 1991; Matsumoto et al. 1993). Interestingly, Hal increased NADPH‐d activity in both the DL and DM regions. Increased NADPH‐d activity was also observed following the intermediate dose of MCP in the DL region and after the higher dose in the DM region. Treatment with D2R blockers produced no significant changes in nNOS/NADPH‐d activity in the NAc, but there was an increase in NADPH‐d activity in the VL region at the lowest cataleptic dose of Hal and the intermediate cataleptic dose of MCP. Notably, with MCP, the intermediate cataleptic dose (8 mg/kg) increased nNOS/NADPH‐d activity in the lateral striatal areas (DL + VL). In the same context, Hal and MCP did not appear to affect NAc responses, as no changes were observed in nNOS immunoreactivity and NADPH‐d activity following their administration. In addition, the increased NADPH‐d activity in the NAc shell region in response to L‐NOARG could be associated with the limbic component of catalepsy (Prinssen and Bemelmans 1994).

### 4.4 Integrative and Mechanistic Interpretation

The NAc shell region has been identified as a possible locus of action for the therapeutic effects of antipsychotics (Deutch et al. 1992), consistent with the changes reported in this area following Hal and L‐NOARG but not MCP for c‐Fos‐IR. The NAc is innervated by the medial ventral tegmental area (Mccutcheon et al. 2019), an important locus of antipsychotic action (Deutch et al. 1992; Mccutcheon et al. 2019), and is considered a promising target for therapeutic interventions in schizophrenia, particularly through the modulation of glutamate‐induced excitotoxicity and NO/pro‐inflammatory cytokine release by activated microglia in striatum and NAc (L. Zhang and Zhao 2014). Although NOS inhibitors reduce NOS activity, compensatory NO synthesis reflected by increased NADPH‐d activity may result from free S‐nitrosothiols (SNOs) or nitrosated proteins, including several NOS isoforms (Seckler et al. 2020). Such compensatory mechanisms have also been observed in sensitization protocols involving drugs of abuse, such as cocaine (Santos et al. 2015). Therapeutic strategies targeting NO disturbances have emerged from evidence linking altered NO signaling to schizophrenia. In this context, antipsychotics can modulate NO metabolism and help restore NMDA receptor function, particularly through adenosine A2A–D2R heteromer interactions (Nasyrova et al. 2015). Although MCP antagonizes striatal D2Rs, it is considered to lack antipsychotic efficacy (Frederick and Schnell 2003). Our findings suggest that altered NO activity may represent a shared mechanism contributing to EPS across different pharmacological manipulations, as NO diffuses widely and plays a central role in motor regulation (West et al. 2002).

In our analysis, linear regression was an important tool, as it allowed us to predict the relationship between behavioral activity and histological results (see Table 2). The analyses revealed that c‐Fos‐IR and nitric oxide activity (NADPH‐d, nNOS) are strong predictors of striatum‐mediated motor behaviors. In Hal‐treated mice, catalepsy was predicted by DL c‐Fos and NADPH‐d (~68% variance), whereas Rota‐rod performance involved more distributed predictors (~88% variance). In MCP‐treated mice, models explained ~68% of catalepsy and 60% of Rota‐rod variance, with DL NADPH‐d and VL nNOS as key predictors of catalepsy, and DM/NAc activity predicting Rota‐rod performance. For L‐NOARG, catalepsy was explained by DM/NAc shell c‐Fos and dorsal striatum nNOS (~71% variance), whereas Rota‐rod performance was predicted by NAc shell c‐Fos and DM NADPH‐d (~55% variance).

Taken together, these findings indicate that striatal nitric oxide signaling and cellular activation in specific subregions—particularly the DL, DM, and NAc—are key modulators of motor impairments induced by dopaminergic blockers and NO‐related pharmacological inhibitors. Although this is not a novel observation, our regression results indicate a functional separation of striatal subregions, highlighting DL NADPH‐d activity linked to catalepsy and DM nNOS/NADPH‐d activity linked to Rota‐rod performance.

Additionally, these results indicate that catalepsy and motor dysfunction induced by D2R blockers are significantly correlated with increased c‐Fos‐IR in the DL striatum, a region implicated in the development of EPS. We also observed that, in this region, the increase in nNOS induced by D2R antagonists positively correlates with NADPH‐d activity, suggesting a possible elevation of NO levels in the striatum during this blockade.

In short, the striatum contains three functional domains—sensorimotor, associative, and limbic—corresponding roughly to the DL, DM, and ventral regions, respectively (Hunnicutt et al. 2016). Projectome studies suggest that the DM striatum is a key associative hub involved in integrating cognitive, proprioceptive, and sensory inputs as well as in early motor‐sequence formation (Hunnicutt et al. 2016; Rotariu et al. 2025). Our findings suggest that D2R antagonists and L‐NOARG primarily act in the DM region. While Hal and MCP impaired motor coordination in the Rota‐rod test, L‐NOARG did not, despite inducing cataleptic responses. This dissociation indicates that motor balance is preserved with L‐NOARG, highlighting a region‐specific effect on striatal circuits rather than a global impairment of motor function.

Overall, although the hypothesis of DARs/glutamate receptors/NO interaction in catalepsy or motor dysfunction has been extensively discussed (Araki et al. 2001; Bishnoi et al. 2009; Connop et al. 1995; E. A. Del Bel et al. 2005; Fontana et al. 2005; Krzaścik and Kostowski 1997; Padovan‐Neto et al. 2009), only a few studies have investigated catalepsy induced by D2R antagonists through modulation of nNOS activity with NADPH‐d in specific striatal regions (Del‐Bel et al. 2010; Del‐Bel and Guimarães 2000; Prieto et al. 2019), as examined here across a range of acute doses. Biochemically, L‐NOARG and L‐NAME act as false substrates for NOS enzymes by interacting with arginine‐binding sites, requiring both an active enzyme and NADPH to achieve full inhibition (Víteček et al. 2012). Thus, it cannot be ruled out that the doses used in the present study were insufficient to fully inhibit nNOS in the dorsal striatum, or that they produced only a partial and weak reversible inhibitory effect on nNOS. Additionally, it is important to note that increased NADPH‐d activity reflects the presence of NO‐related factors rather than the amount of NOS enzyme in fixed tissue (Seckler et al. 2020); therefore, both NOS‐IR and NADPH‐d activity should be assessed.

As points of consideration, our results suggest that (i) modulation of nitric oxide activity in the dorsal striatum—mainly in the DL region—contributes to the pathogenesis of early EPS, such as catalepsy; (ii) nNOS/NADPH‐d activity in the DM region is involved in Rota‐rod performance or motor coordination; (iii) although NADPH‐d is widely known as a specific marker for neurons producing nitric oxide, an increase in NADPH‐d activity following free SNOs or nitrosated proteins, including NOS itself and its isoforms, cannot be excluded (Seckler et al. 2020); and (iv) importantly, striatal c‐Fos cannot be used as the unique predictor of catalepsy.

Future studies should assess whether specific nNOS inhibitors (e.g., NPA, l‐VNIO, and 7‐NI) reduce c‐Fos and nNOS/NADPH‐d coexpression in dorsal striatum—where both markers are enriched in the matrix—and whether altered nNOS/NADPH‐d activity in specific subregions contributes to side effects such as tardive dyskinesia (Lau et al. 2003). This effect may be correlated with NMDA receptor–driven Ca^2+^ increases in D1‐ and D2‐MSNs, along with glutamatergic input to interneurons that enhance nNOS activity (Calabresi et al. 2014).

Another point is that establishing an appropriate dose–response curve, avoiding plateau or sedative doses as seen with Hal or MCP, may help clarify the relationship between NOS/NADPH‐d activity, catalepsy, and motor balance impairment in motor control in each region. Hal‐induced c‐Fos also depends on synergistic NMDA and A2a (adenosine) receptor activation (Onimus et al. 2022), and A2aR stimulation can produce sedation (Hong et al. 2005). This is supported by the absence of a sedative effect with L‐NOARG in the Rota‐rod test, which appears to primarily modulate dopamine function.

Finally, our results suggest that EPS side effects at certain acute dosages can be attributed to NADPH‐d activity in the dorsal striatum, based on the premise that NO signaling modulates D2R‐related motor pathways by regulating striatal excitability and NMDA receptor coupling.

## 5 Conclusion

Catalepsy and motor balance deficits can arise following the acute administration of D2R blockers, with increased c‐Fos‐IR observed in the dorsal striatum after Hal and in the DL striatum following therapeutic doses of MCP. Both types of D2R antagonists led to increased NADPH‐d activity in the dorsal striatum. In contrast, higher cataleptic doses of L‐NOARG elicited a reduction of c‐Fos‐IR in the DL striatum and NAc but also resulted in increased NADPH‐d activity in the dorsal striatum. Furthermore, acute EPS side effects were significantly correlated with NADPH‐d activity in the dorsal striatum. Regression analysis revealed that the most important predictor in our models was NADPH‐d activity in the DL region for catalepsy and nNOS/NADPH activity in the DM region for Rota‐rod performance. These findings indicate that NO signaling plays a crucial role in EPS and motor deficits, facilitating acute EPS through increased NADPH‐d activity in the dorsolateral striatum, whereas nNOS/NADPH‐d activity in the dorsomedial region contributes to motor coordination. This functional segregation of striatal subregions underscores the nuanced role of NO in mediating EPS and motor deficits.

## Author Contributions

**Sonia Guerrero Prieto:** conceptualization, data curation, investigation, methodology, writing – original draft, writing – review and editing. **Victor Ricardo C. Torres da Silva:** data curation, formal analysis, investigation, methodology, visualization, writing – original draft, writing – review and editing. **Maria Camila Almeida:** funding acquisition, resources, writing – original draft, writing – review and editing. **Marcela B. Echeverry:** conceptualization, formal analysis, funding acquisition, investigation, methodology, resources, visualization, writing – original draft, writing – review and editing.

## Funding

This work was supported by the Coordenação de Aperfeiçoamento de Pessoal de Nível Superior, (No. 001) Universidad de Santander (No. 010‐11), and Fundação de Amparo à Pesquisa do Estado de São Paulo (No. 2015/02991‐0).

## Ethics Statement

Ethics Committee on Animal Use (CEUA‐UFABC) protocol No. 1362060218.

## Conflicts of Interest

The authors declare no conflicts of interest.

## Supporting information


**Table S1:** Behavioral ANOVA results.
**Table S2:** Histochemistry ANOVA results.

## Data Availability

The data that support the findings of this study are openly available in Zenodo at https://doi.org/10.5281/zenodo.17872413, reference number zenodo.17872413.

## References

[ejn70386-bib-0001] Agovic, M. S. , E. Yablonsky‐Alter , T. I. Lidsky , and S. P. Banerjee . 2008. “Mechanisms for Metoclopramide‐Mediated Sensitization and Haloperidol‐Induced Catalepsy in Rats.” European Journal of Pharmacology 587, no. 1–3: 181–186. 10.1016/j.ejphar.2008.03.056. 18457824

[ejn70386-bib-0002] Ahmad Khan, H. , S. Al Deeb , K. Al Moutaery , and M. Tariq . 2004. “Metoclopramide Attenuates Iminodipropionitrile‐Induced Oxidative Stress and Neurobehavioral Toxicity in Rats.” Pharmacology Biochemistry and Behavior 79, no. 3: 555–561. 10.1016/j.pbb.2004.09.006. 15582028

[ejn70386-bib-0003] Ahtee, L. 1975a. “Effect of Drugs on Metoclopramide Induced Catalepsy and Increase in Striatal Homovanillic Acid Content.” British Journal of Pharmacology 53, no. 3: 1975. PMC1666406 1169083

[ejn70386-bib-0004] Ahtee, L. 1975b. “Inhibition Byapomorphine of the Metoclopramide‐Induced.” British Journal of Pharmacology 55: 381–385. 1239323 10.1111/j.1476-5381.1975.tb06941.x PMC1666697

[ejn70386-bib-0005] Ahtee, L. , and G. Buncombe . 1974. “Metoclopramide Induces Catalepsy and Increases Stiatal Homovanillic Acid Content in Mice.” Acta Pharmacology and Toxicology (Copenh) 35: 429–432. 10.1111/j.1600-0773.1974.tb00764.x 4479719

[ejn70386-bib-0006] Al‐Saffar, A. , H. Lennernäs , and P. M. Hellström . 2019. “Gastroparesis, Metoclopramide, and Tardive Dyskinesia: Risk Revisited.” Neurogastroenterology and Motility 31, no. 11: e13617. 10.1111/nmo.13617. 31050085

[ejn70386-bib-0007] Araki, T. , H. Mizutani , M. Matsubara , Y. Imai , M. Mizugaki , and Y. Itoyama . 2001. “Nitric Oxide Synthase Inhibitors Cause Motor Deficits in Mice.” European Neuropsychopharmacology 11: 125–133. 10.1016/s0924-977x(01)00077-3. 11313158

[ejn70386-bib-0008] Baker, T. W. , M. M. Florczynski , and R. J. Beninger . 2014. “Differential Effects of Clozapine, Metoclopramide, Haloperidol and Risperidone on Acquisition and Performance of Operant Responding in Rats.” Psychopharmacology 232, no. 9: 1535–1543. 10.1007/s00213-014-3789-6. 25381749

[ejn70386-bib-0009] Banasikowski, T. J. , and R. J. Beninger . 2012. “Haloperidol Conditioned Catalepsy in Rats: A Possible Role for D 1‐Like Receptors.” International Journal of Neuropsychopharmacology 15, no. 10: 1525–1534. 10.1017/S1461145711001696. 22093169

[ejn70386-bib-0010] Bateup, H. S. , P. Svenningsson , M. Kuroiwa , et al. 2008. “Cell Type–Specific Regulation of DARPP‐32 Phosphorylation by Psychostimulant and Antipsychotic Drugs.” Nature Neuroscience 11, no. 8: 932–939. 10.1038/nn.2153. 18622401 PMC2737705

[ejn70386-bib-0011] Batista, L. A. , T. G. Viana , V. T. Silveira , D. C. Aguiar , and F. A. Moreira . 2016. “Effects of Aripiprazole on Caffeine‐Induced Hyperlocomotion and Neural Activation in the Striatum.” Naunyn‐Schmiedeberg's Archives of Pharmacology 389, no. 1: 11–16. 10.1007/s00210-015-1170-x. 26319049

[ejn70386-bib-0012] Bishnoi, M. , K. Chopra , and S. K. Kulkarni . 2009. “Pharmacology, Biochemistry and Behavior Co‐Administration of Nitric Oxide (NO) Donors Prevents Haloperidol‐Induced Orofacial Dyskinesia, Oxidative Damage and Change in Striatal Dopamine Levels.” Pharmacology, Biochemistry and Behavior 91, no. 3: 423–429. 10.1016/j.pbb.2008.08.021. 18789960

[ejn70386-bib-0013] Bonito‐Oliva, A. , M. Feyder , and G. Fisone . 2011. “Deciphering the Actions of Antiparkinsonian and Antipsychotic Drugs on cAMP/DARPP‐32 Signaling.” Frontiers in Neuroanatomy 5, no. July: 38. 10.3389/fnana.2011.00038. 21808606 PMC3136733

[ejn70386-bib-0014] Brenman, J. E. , D. S. Chao , S. H. Gee , et al. 1996. “Interaction of Nitric Oxide Synthase With the Postsynaptic Density Protein PSD‐95 and α1‐Syntrophin Mediated by PDZ Domains.” Cell 84, no. 5: 757–767. 10.1016/S0092-8674(00)81053-3. 8625413

[ejn70386-bib-0091] Brimblecombe, K. R. , and S. J. Cragg . 2017. “The Striosome and Matrix Compartments of the Striatum: A Path Through the Labyrinth from Neurochemistry toward Function.” ACS Chemical Neuroscience 8, no. 2: 235–242. 10.1021/acschemneuro.6b00333. 27977131

[ejn70386-bib-0090] Bubser, M. , and A. Y. Deutch . 2002. “Differential Effects of Typical and Atypical Antipsychotic Drugs on Striosome and Matrix Compartments of the Striatum.” European Journal of Neuroscience 15, no. 4: 713–720. 10.1046/j.1460-9568.2002.01903.x. 11886451

[ejn70386-bib-0015] Burton, A. C. , K. Nakamura , and M. R. Roesch . 2015. “From Ventral‐Medial to Dorsal‐Lateral Striatum: Neural Correlates of Reward‐Guided Decision‐Making.” Neurobiology of Learning and Memory 117: 51–59. 10.1016/j.nlm.2014.05.003. 24858182 PMC4240773

[ejn70386-bib-0085] Calabresi, P. , P. Gubellini , D. Centonze , et al. 1999. “A Critical Role of the Nitric Oxide/cGMP Pathway in Corticostriatal Long‐Term Depression.” Journal of Neuroscience 19, no. 7: 2489–2499. 10.1523/JNEUROSCI.19-07-02489.1999. 10087063 PMC6786075

[ejn70386-bib-0086] Calabresi, P. , B. Picconi , A. Tozzi , V. Ghiglieri , and M. Di Filippo . 2014. “Direct and Indirect Pathways of Basal Ganglia: A Critical Reappraisal.” Nature Neuroscience 17, no. 8: 1022–1030. 10.1038/nn.3743. 25065439

[ejn70386-bib-0016] Casteels‐Van Daele, M. , J. Jaeken , P. Van Der Schueren , A. Zimmerman , and P. Van Den Bon . 1970. “Dystonic Reactions in Children Caused by Metoclopramide.” Archives of Disease in Childhood 45, no. 239: 130–133. 10.1136/adc.45.239.130. 5440179 PMC2020385

[ejn70386-bib-0017] Connop, B. P. , R. J. Boegman , K. Jhamandas , and R. J. Beninger . 1995. “Excitotoxic Action of NMDA Agonists on Nigrostriatal Dopaminergic Neurons: Modulation by Inhibition of Nitric Oxide Synthesis.” Brain Research 676: 124–132. 7540931 10.1016/0006-8993(95)00103-w

[ejn70386-bib-0088] Dawson, T. M. , D. S. Bredt , M. Fotuhi , P. M. Hwang , and S. H. Snyder . 1991. “Nitric Oxide Synthase and Neuronal NADPH Diaphorase Are Identical in Brain and Peripheral Tissues.” Proceedings of the National Academy of Sciences of the United States of America 88, no. 17: 7797–7801. 10.1073/pnas.88.17.7797. 1715581 PMC52390

[ejn70386-bib-0018] de Bartolomeis, A. , E. F. Buonaguro , G. Latte , and R. Rossi . 2017. “Immediate‐Early Genes Modulation by Antipsychotics: Translational Implications for a Putative Gateway to Drug‐Induced Long‐Term Brain Changes.” Frontiers in Behavioral Neuroscience 11, no. December: 1–24. 10.3389/fnbeh.2017.00240. 29321734 PMC5732183

[ejn70386-bib-0019] De Oliveira, C. L. , E. A. Del Bel , and F. S. Guimarães . 1996. “Effects of L‐NOARG on Plus‐Maze Performance in Rats.” Pharmacology Biochemistry and Behavior 56, no. 1: 55–59. 10.1016/S0091-3057(96)00156-6. 8981609

[ejn70386-bib-0020] Dekundy, A. , M. Lundblad , W. Danysz , and M. A. Cenci . 2007. “Modulation of l‐DOPA‐Induced Abnormal Involuntary Movements by Clinically Tested Compounds: Further Validation of the Rat Dyskinesia Model.” Behavioural Brain Research 179, no. 1: 76–89. 10.1016/j.bbr.2007.01.013. 17306893

[ejn70386-bib-0022] Del Bel, E. A. , C. A. Da Silva , F. S. Guimarães , and M. Bermúdez‐Echeverry . 2004. “Catalepsy Induced by Intra‐Striatal Administration of Nitric Oxide Synthase Inhibitors in Rats.” European Journal of Pharmacology 485, no. 1–3: 175–181. 10.1016/j.ejphar.2003.11.071. 14757138

[ejn70386-bib-0023] Del Bel, E. A. , F. S. Guimarães , M. Bermũdez‐Echeverry , et al. 2005. “Role of Nitric Oxide on Motor Behavior.” Cellular and Molecular Neurobiology 25, no. 2: 371–392. 10.1007/s10571-005-3065-8. 16047547 PMC11529539

[ejn70386-bib-0021] Del Bel, E. , A. Souza , F. Guimarães , C. Da‐Silva , and L. Nucci‐da‐Silva . 2002. “Motor Effects of Acute and Chronic Inhibition of Nitric Oxide Synthesis in Mice.” Psychopharmacology 161, no. 1: 32–37. 10.1007/s00213-002-1009-2. 11967628

[ejn70386-bib-0024] Del‐Bel, E. A. , and F. S. Guimarães . 2000. “Sub‐Chronic Inhibition of Nitric‐Oxide Synthesis Modifies Haloperidol‐Induced Catalepsy and the Number of NADPH‐Diaphorase Neurons in Mice.” Psychopharmacology 147: 356–361. 10672628 10.1007/s002130050003

[ejn70386-bib-0025] Del‐Bel, E. A. , F. S. Guimarães , S. R. L. Joca , M. B. Echeverry , and F. R. Ferreira . 2010. “Tolerance to the Cataleptic Effect That Follows Repeated Nitric Oxide Synthase Inhibition May Be Related to Functional Enzymatic Recovery.” Journal of Psychopharmacology 24, no. 3: 397–405. 10.1177/0269881108097717. 18838497

[ejn70386-bib-0087] DeLong, M. R. , and T. Wichmann . 2007. “Circuits and Circuit Disorders of the Basal Ganglia.” Archives of Neurology 64: 20–24. 10.1001/archneur.64.1.20. 17210805

[ejn70386-bib-0026] Deutch, A. Y. , M. C. Lee , and J. Iadarolat . 1992. “Regionally Specific Effects of Atypical Antipsychotic Drugs on Striatal Fos Expression: The Nucleus Accumbens Shell as a Locus of Antipsychotic Action.” Molecular and Cellular Neurosciences 3: 332–341. 19912876 10.1016/1044-7431(92)90030-6

[ejn70386-bib-0027] Dunstan, R. , and C. L. Broekkamp . 1981. “Involvement of Caudate Nucleus, Amygdala or Reticular Formation in Neuroleptic and Narcotic Catalepsy.” Pharmacology Biochemistry and Behavior 14: 169–174. 7193882 10.1016/0091-3057(81)90239-2

[ejn70386-bib-0028] Echeverry, M. B. , F. S. Guimarães , and E. Del Bel . 2004. “Acute and Delayed Restraint Stress‐Induced Changes in Nitric Oxide Producing Neurons in Limbic Regions.” Neuroscience 125, no. 4: 981–993. 10.1016/j.neuroscience.2003.12.046. 15120858

[ejn70386-bib-0029] Echeverry, M. B. , M. L. Salgado , F. R. Ferreira , C. da‐Silva , and E. Del Bel . 2007. “Intracerebroventricular Administration of Nitric Oxide‐Sensitive Guanylyl Cyclase Inhibitors Induces Catalepsy in Mice.” Psychopharmacology 194, no. 2: 271–278. 10.1007/s00213-007-0834-8. 17593355

[ejn70386-bib-0030] Fontana, L. , A. Souza , E. A. Del‐Bel , and R. M. W. Oliveira . 2005. “Ginkgo Biloba Leaf Extract (EGb 761) Enhances Catalepsy Induced by Haloperidol and L‐Nitroarginine in Mice.” Brazilian Journal and Biological Research 38: 1649–1654. 10.1590/s0100-879x2005001100012 16258634

[ejn70386-bib-0097] Frederick, M. , and F. M. Schnell . 2003. “Chemotherapy‐Induced Nausea and Vomiting: The Importance of Acute Antiemetic Control.” Oncologist 8, no. 2: 187–198. 10.1634/theoncologist.8-2-187. 12697943

[ejn70386-bib-0031] Friedman, B. W. , L. Mulvey , D. Esses , et al. 2011. “Metoclopramide for Acute Migraine: A Dose‐Finding Randomized Clinical Trial.” Annals of Emergency Medicine 57, no. 5: 475–482.e1. 10.1016/j.annemergmed.2010.11.023. 21227540 PMC3341930

[ejn70386-bib-0032] Gupta, N. , and D. Basu . 2001. “Does Risperidone Reduce Concomitant Substance Abuse in Cases of Schizophrenia?” Canadian Journal of Psychiatry 46, no. 9: 862–863. 10.1177/070674370104600919 11761640

[ejn70386-bib-0033] Hartgraves, S. L. , and P. H. Kelly . 1984. “Role of Mesencephalic Reticular Formation in Cholinergic‐Induced Catalepsy and Anticholinergic Reversal of Neuroleptic‐Induced Catalepsy.” Brain Research 307: 47–54. 6540616 10.1016/0006-8993(84)90458-x

[ejn70386-bib-0034] Hiroi, N. , and A. M. Graybiel . 1996. “Atypical and Typical Neuroleptic Treatments Induce Distinct Programs of Transcription Factor Expression in the Striatum.” Journal of Comparative Neurology 374, no. 1: 70–83. 10.1002/(SICI)1096-9861(19961007)374:1<70::AID-CNE5>3.0.CO;2-K. 8891947

[ejn70386-bib-0102] Hong, Z. Y. , Z. L. Huang , W. M. Qu , N. Eguchi , Y. Urade , and O. Hayaishi . 2005. “An Adenosine A Receptor Agonist Induces Sleep by Increasing GABA Release in the Tuberomammillary Nucleus to Inhibit Histaminergic Systems in Rats.” Journal of Neurochemistry 92, no. 6: 1542–1549. 10.1111/j.1471-4159.2004.02991.x. 15748171

[ejn70386-bib-0089] Hope, B. T. , G. J. Michael , K. M. Knigge , and S. R. Vincent . 1991. “Neuronal NADPH Diaphorase Is a Nitric Oxide Synthase.” Proceedings of the National Academy of Sciences of the United States of America 88, no. 7: 2811–2814. 10.1073/pnas.88.7.2811. 1707173 PMC51329

[ejn70386-bib-0035] Hu, J. , J. H. Lee , and E. E. Ei‐fakahany . 1994. “Inhibition of Neuronal Nitric Oxide Synthase by Antipsychotic Drugs.” Psychopharmacology 114: 161–166. 7531351 10.1007/BF02245458

[ejn70386-bib-0099] Hunnicutt, B. J. , B. C. Jongbloets , W. T. Birdsong , K. J. Gertz , H. Zhong , and T. Mao . 2016. “A Comprehensive Excitatory Input Map of the Striatum Reveals Novel Functional Organization.” Elife 28, no. 5: e19103. 10.7554/eLife.19103. PMC5207773 27892854

[ejn70386-bib-0036] Imamura, Y. , S. Harada , Y. Okano , T. Miyata , and M. Otagiri . 1988. “The Effect of Metoclopramide on the Absorption of Oral Controlled Release Morphine.” British Journal of Clinical Pharmacology 25, no. 4: 518–521. 10.1111/j.1365-2125.1988.tb03338.x. 3382595 PMC1387816

[ejn70386-bib-0037] Issy, A. C. , and E. A. Del Bel . 2014. “7‐Nitroindazole Blocks the Prepulse Inhibition Disruption and c‐Fos Increase Induced by Methylphenidate.” Behavioural Brain Research 262: 74–83. 10.1016/j.bbr.2013.12.042. 24406716

[ejn70386-bib-0038] Issy, A. C. , M. Lazzarini , R. E. Szawka , R. O. G. Carolino , J. A. Anselmo‐franci , and E. A. Bel . 2011. “Nitric Oxide Synthase Inhibitors Improve Prepulse Inhibition Responses of Wistar Rats.” Behavioural Brain Research 217, no. 2: 416–423. 10.1016/j.bbr.2010.11.016. 21074571

[ejn70386-bib-0039] Iwanami, S. , M. Takashima , Y. Hirata , O. Hasegawa , and S. Usuda . 1981. “Synthesis and Neuroleptic Activity of Benzamides. cis‐N‐(1‐Benzyl‐2‐methylpyrrolidin‐3‐yl)‐5‐chloro‐2‐met hoxy‐4‐(met hylamino)benzamide and Related Compounds.” Journal of Medicinal Chemistry 24, no. 1: 1224–1230. 6120234 10.1021/jm00142a019

[ejn70386-bib-0040] Joshi, R. S. , R. Quadros , M. Drumm , R. Ain , and M. M. Panicker . 2017. “Sedative Effect of Clozapine Is a Function of 5‐HT2A and Environmental Novelty.” European Neuropsychopharmacology 27, no. 1: 70–81. 10.1016/j.euroneuro.2016.10.007. 27955831

[ejn70386-bib-0041] Kiss, J. P. , and E. S. Vizi . 2001. “Nitric Oxide: A Novel Link Between Synaptic and Nonsynaptic Transmission.” Trends in Neurosciences 24, no. 4: 211–215. 10.1016/S0166-2236(00)01745-8. 11250004

[ejn70386-bib-0042] Krzaścik, P. , and W. Kostowski . 1997. “Nitric Oxide Donors Antagonize N‐Nitro‐L‐Arginine and Haloperidol Catalepsy: Potential Implication for the Treatment of Parkinsonism?” Polish Journal of Pharmacology 49, no. 4: 263–266. 9437770

[ejn70386-bib-0043] Lau, Y. S. , E. Petroske , G. E. Meredith , and J. Q. Wang . 2003. “Elevated Neuronal Nitric Oxide Synthase Expression in Chronic Haloperidol‐Treated Rats.” Neuropharmacology 45, no. 7: 986–994. 10.1016/S0028-3908(03)00314-9. 14573391

[ejn70386-bib-0083] Mallet, N. , C. Le Moine , S. Charpier , and F. Gonon . 2005. “Feedforward Inhibition of Projection Neurons by Fast‐Spiking GABA Interneurons in the Rat Striatum in Vivo.” Journal of Neuroscience 25, no. 15: 3857–3869. 10.1523/JNEUROSCI.5027-04.2005. 15829638 PMC6724938

[ejn70386-bib-0044] Marras, R. A. , A. P. Martins , E. A. Del Bel , and F. S. Guimarães . 1995. “L‐NOARG, an Inhibitor of Nitric Oxide Synthase, Induces Catalepsy in Mice.” Neuroreport 7, no. 1: 158–160. http://europepmc.org/abstract/MED/8742441. 8742441

[ejn70386-bib-0045] Matsumoto, T. , M. Nakane , J. Pollock , J. Kuk , and U. Forstermann . 1993. “Diaphorase Activity Is Only Seen After Exposure of the Tissue to Fixative.” Neuroscience Letters 155: 61–64. 7689718 10.1016/0304-3940(93)90673-9

[ejn70386-bib-0046] Mccutcheon, R. A. , A. Abi‐dargham , and O. D. Howes . 2019. “Schizophrenia, Dopamine and the Striatum: From Biology to Symptoms.” Trends in Neurosciences 42, no. 3: 205–220. 10.1016/j.tins.2018.12.004. 30621912 PMC6401206

[ejn70386-bib-0047] Merchant, K. M. , and D. M. Dorsa . 1993. “Differential Induction of Neurotensin and c‐Fos Gene Expression by Typical Versus Atypical Antipsychotics.” Proceedings of the National Academy of Sciences of The United States of America 90, no. 8: 3447–3451. 8097317 10.1073/pnas.90.8.3447 PMC46317

[ejn70386-bib-0048] Miller, L. , and J. Jankovic . 1989. “Metoclopramide‐Induced Movement Disorders: Clinical Findings With a Review of the Literature.” Archives of Internal Medicine 149, no. 11: 2486–2492. 10.1001/archinte.1989.00390110070015. 2684075

[ejn70386-bib-0049] Morris, B. J. , C. S. Simpson , S. Mundell , K. Maceachern , H. M. Johnston , and A. M. Nolan . 1997. “Dynamic Changes in NADPH‐Diaphorase Staining Reflect Activity of Nitric Oxide Synthase: Evidence for a Dopaminergic Regulation of Striatal Nitric Oxide Release.” Neuropharmacology 36, no. 11–12: 1589–1599. 10.1016/S0028-3908(97)00159-7. 9517430

[ejn70386-bib-0096] Nasyrova, R. F. , D. V. Ivashchenko , M. V. Ivanov , and N. G. Neznanov . 2015. “Role of Nitric Oxide and Related Molecules in Schizophrenia Pathogenesis: Biochemical, Genetic and Clinical Aspects.” Frontiers in Physiology 6: 139. 10.3389/fphys.2015.00139. 26029110 PMC4426711

[ejn70386-bib-0093] Nel, A. , and B. H. Harvey . 2003. “Haloperidol‐Induced Dyskinesia Associated with Striatal NO Synthase Inhibition: Reversal with Olanzapine.” Behavioural Pharmacology 14: 251–255. 10.1097/00008877-200305000-00010. 12799528

[ejn70386-bib-0050] Nguyen, T. V. , B. E. Kosofsky , R. Birnbaum , B. M. Cohen , and S. E. Hyman . 1992. “Differential Expression of c‐Fos and Zif268 in Rat Striatum After Haloperidol, Clozapine, and Amphetamine.” Proceedings of the National Academy of Sciences of the United States of America 89, no. 10: 4270–4274. 10.1073/pnas.89.10.4270. 1374894 PMC49063

[ejn70386-bib-0051] Nucci‐Da‐Silva, L. P. , F. S. Guimarães , and E. A. Del Bel . 1999. “Serotonin Modulation of Catalepsy Induced by N(G)‐Nitro‐L‐Arginine in Mice.” European Journal of Pharmacology 379, no. 1: 47–52. 10.1016/S0014-2999(99)00493-8. 10499370

[ejn70386-bib-0101] Onimus, O. , E. Valjent , G. Fisone , and G. Gangarossa . 2022. “Haloperidol‐Induced Immediate Early Genes in Striatopallidal Neurons Requires the Converging Action of cAMP/PKA/DARPP‐32 and mTOR Pathways.” International Journal of Molecular Sciences 23, no. 19: 11637. 10.3390/ijms231911637. 36232936 PMC9569967

[ejn70386-bib-0052] Padovan‐Neto, F. E. , R. Cavalcanti‐Kiwiatkoviski , R. O. G. Carolino , J. Anselmo‐Franci , and E. Del Bel . 2015. “Effects of Prolonged Neuronal Nitric Oxide Synthase Inhibition on the Development and Expression of l‐DOPA‐Induced Dyskinesia in 6‐OHDA‐Lesioned Rats.” Neuropharmacology 89: 87–99. 10.1016/j.neuropharm.2014.08.019. 25196732

[ejn70386-bib-0053] Padovan‐Neto, F. E. , M. B. Echeverry , V. Tumas , and E. Del‐Bel . 2009. “Nitric Oxide Synthase Inhibition Attenuates L‐DOPA‐Induced Dyskinesias in a Rodent Model of Parkinson's Disease.” Neuroscience 159, no. 3: 927–935. 10.1016/j.neuroscience.2009.01.034. 19302833

[ejn70386-bib-0054] Paxinos, G. , and K. Franklin . 2008. The Mouse Brain in Stereotaxic Coordinates. In Academic Press.

[ejn70386-bib-0055] Pinna, A. , J. Wardas , A. Cozzolino , and M. Morelli . 1999. “Involvement of Adenosine A(2A) Receptors in the Induction of c‐Fos Expression by Clozapine and Haloperidol.” Neuropsychopharmacology 20, no. 1: 44–51. 10.1016/S0893-133X(98)00051-7. 9885784

[ejn70386-bib-0056] Popeski, N. , and B. Woodside . 2001. “Effect of Nitric Oxide Synthase Inhibition on Fos Expression in the Hypothalamus of Female Rats Following Central Oxytocin and Systemic Urethane Administration.” Journal of Neuroendocrinology 13, no. 10: 596–607. 11442774 10.1046/j.1365-2826.2001.00673.x

[ejn70386-bib-0057] Prieto, S. C. , J. C. S. Silva , M. O. De Lima , M. C. Almeida , and M. B. Echeverry . 2019. “Cross‐Tolerance Between Nitric Oxide Synthase Inhibition and Atypical Antipsychotics Modify Nicotinamide‐Adenine‐Dinucleotide Phosphate‐Diaphorase Activity in Mouse Lateral Striatum.” Behavioural Pharmacology 30, no. 1: 67–78. 10.1097/FBP.0000000000000406. 29664745

[ejn70386-bib-0058] Prinssen, E. P. M. , and F. J. Bemelmans . 1994. “Evidence for a Role of the Shell of the Nucleus Behavior of Freely Moving Rats in Oral.” Journal of Neuroscience 74, no. March: 1555–1562. 10.1523/JNEUROSCI.14-03-01555.1994 PMC6577530 7907364

[ejn70386-bib-0059] Reagan‐Shaw, S. , M. Nihal , and N. Ahmad . 2007. “Dose Translation From Animal to Human Studies Revisited.” FASEB Journal 22, no. 3: 659–661. 10.1096/fj.07-9574lsf. 17942826

[ejn70386-bib-0060] Robertson, G. S. , H. Matsumura , and H. C. Fibiger . 1994. “Induction Patterns of Fos‐Like Immunoreactivity in the Forebrain as Predictors of Atypical Antipsychotic Activity.” Journal of Pharmacology and Experimental Therapeutics 271, no. 2: 1058–1066. 7965768

[ejn70386-bib-0061] Robertson, G. S. , S. R. Vincent , and H. C. Fibiger . 1992. “D1 and D2 Dopamine Receptors Differentially Regulate c‐Fos Expression in Striatonigral and Striatopallidal Neurons.” Neuroscience 49, no. 2: 285–296. 10.1016/0306-4522(92)90096-K. 1359451

[ejn70386-bib-0062] Rogue, P. , and G. Vincendon . 1992. “Dopamine D2 Receptor Antagonists Induce Immediate Early Genes in the Rat Striatum.” Brain Research Bulletin 29, no. 3–4: 469–472. 10.1016/0361-9230(92)90084-B. 1356602

[ejn70386-bib-0063] Romanova, E. V. , S. S. Rubakhin , J. R. Ossyra , et al. 2016. “Differential Peptidomics Assessment of Strain and Age Differences in Mice in Response to Acute Cocaine Administration.” Journal of Neurochemistry 135, no. 5: 1038–1048. 10.1111/jnc.13265. PMC4804715 26223348

[ejn70386-bib-0100] Rotariu, S. , G. Zalcman , N. Badreddine , et al. 2025. “Somatostatin Interneurons Select Dorsomedial Striatal Representations of the Initial Motor Learning Phase.” Cell Reports 44, no. 5: 115670. 10.1016/j.celrep.2025.115670. 40333184

[ejn70386-bib-0092] Saka, E. , M. Iadarola , D. J. Fitzgerald , and A. M. Graybiel . 2002. “Local Circuit Neurons in the Striatum Regulate Neural and Behavioral Responses to Dopaminergic Stimulation.” Proceedings of the National Academy of Sciences of the United States of America 99: 9004–9009. 10.1073/pnas.132212499. 12060713 PMC124413

[ejn70386-bib-0064] Sammut, S. , K. E. Bray , and A. R. West . 2007. “Dopamine D2 Receptor‐Dependent Modulation of Striatal NO Synthase Activity.” Psychopharmacology 191, no. 3: 793–803. 10.1007/s00213-006-0681-z. 17206493

[ejn70386-bib-0065] Sanberg, P. R. , M. D. Bunsey , M. Giordano , and A. B. Norman . 1988. “The Catalepsy Test: Its Ups and Downs.” Behavioral Neuroscience 102, no. 5: 748–759. 10.1037/0735-7044.102.5.748. 2904271

[ejn70386-bib-0095] Santos, A. I. , A. Martínez‐Ruiz , and I. M. Araújo . 2015. “S‐Nitrosation and Neuronal Plasticity.” British Journal of Pharmacology 172, no. 6: 1468–1478. 10.1111/bph.12827. 24962517 PMC4369257

[ejn70386-bib-0066] Schatz, D. , V. Kaufmann , R. Schuligoi , C. Humpel , and A. Saria . 2000. “3,4‐Methylenedioxymetamphetamine (Ecstasy) Induces c‐Fos‐Like Protein and mRNAin Rat Organotypic Dorsal Striatal Slices.” Synapse 83, no. August 1999: 75–83. 10.1002/(SICI)1098-2396(200004)36:1<75::AID-SYN8>3.0.CO;2-I 10700028

[ejn70386-bib-0067] Sebens, J. B. , T. Koch , G. J. T. Horst , and J. Korf . 1995. “Differential Fos‐Protein Induction in Rat Forebrain Regions After Acute and Long‐Term Haloperidol and Clozapine Treatment.” European Journal of Pharmacology 273, no. 1–2: 175–182. 10.1016/0014-2999(94)00692-Z. 7737311

[ejn70386-bib-0068] Seckler, J. M. , J. Shen , T. H. J. Lewis , et al. 2020. “NADPH Diaphorase Detects S‐Nitrosylated Proteins in Aldehyde‐Treated Biological Tissues.” Scientific Reports 10, no. 1: 1–14. 10.1038/s41598-020-78107-6. 33273578 PMC7713249

[ejn70386-bib-0069] Shilliam, C. S. , and L. A. Dawson . 2005. “The Effect of Clozapine on Extracellular Dopamine Levels in the Shell Subregion of the Rat Nucleus Accumbens Is Reversed Following Chronic Administration: Comparison With a Selective 5‐HT 2C Receptor Antagonist.” Neuropsychopharmacology 30: 372–380. 10.1038/sj.npp.1300591. 15562297

[ejn70386-bib-0070] Silva, M. , D. C. Aguiar , C. R. A. Diniz , F. S. Guimarães , and S. R. L. Joca . 2012. “Neuronal NOS Inhibitor and Conventional Antidepressant Drugs Attenuate Stress‐Induced Fos Expression in Overlapping Brain Regions.” Cellular and Molecular Neurobiology 32, no. 3: 443–453. 10.1007/s10571-011-9775-1. 22120186 PMC11498573

[ejn70386-bib-0071] Sonego, A. B. , F. V. Gomes , E. A. Del Bel , and F. S. Guimaraes . 2016. “Cannabidiol Attenuates Haloperidol‐Induced Catalepsy and c‐Fos Protein Expression in the Dorsolateral Striatum via 5‐HT1A Receptors in Mice.” Behavioural Brain Research 309: 22–28. 10.1016/j.bbr.2016.04.042. 27131780

[ejn70386-bib-0072] Spolidório, P. C. , M. Echeverry , M. Iyomasa , F. Guimarães , and E. Del Bel . 2007. “Anxiolytic Effects Induced by Inhibition of the Nitric Oxide–cGMP Pathway in the Rat Dorsal Hippocampus.” Psychopharmacology 195: 183–192. 10.1007/s00213-007-0890-0. 17661019

[ejn70386-bib-0073] Starr, B. S. , and M. S. Starr . 1987. “Behavioural Interactions Involving D, and D, Dopamine Receptors in Non‐Habituated Mice.” Neuropharmacology 26, no. September: 613–619. 2955244 10.1016/0028-3908(87)90155-9

[ejn70386-bib-0074] Tulchinsky, E. 2000. “Invited Review Fos Family Members: Regulation, Structure and Role in Oncogenic Transformation.” Histology and Histopathology 15: 921–928. 10963134 10.14670/HH-15.921

[ejn70386-bib-0075] Víteček, J. , A. Lojek , G. Valacchi , and L. Kubala . 2012. “Arginine‐Based Inhibitors of Nitric Oxide Synthase: Therapeutic Potential and Challenges.” Mediators of Inflammation 2012: 318087. 10.1155/2012/318087. 22988346 PMC3441039

[ejn70386-bib-0084] Vuillet, J. , L. Kerkerian , P. Kachidian , O. Bosler , and A. Nieoullon . 1989. “Ultrastructural Correlates of Functional Relationships between Nigral Dopaminergic or Cortical Afferent Fibers and Neuropeptide Y‐Containing Neurons in the Rat Striatum.” Neuroscience Letters 100, no. 1‐3: 99–104. 10.1016/0304-3940(89)90667-8. 2761790

[ejn70386-bib-0076] Wadenberg, M. L. G. , A. Soliman , S. C. VanderSpek , and S. Kapur . 2001. “Dopamine D2 Receptor Occupancy Is a Common Mechanism Underlying Animal Models of Antipsychotics and Their Clinical Effects.” Neuropsychopharmacology 25, no. 5: 633–641. 10.1016/S0893-133X(01)00261-5. 11682246

[ejn70386-bib-0077] Weinberg, M. S. , M. Girotti , and R. L. Spencer . 2008. “Restraint‐Induced Fra‐2 and c‐Fos Expression in the Rat Forebrain: Relationship to Stress Duration.” In Situ 150, no. 2: 478–486. 10.1016/j.neuroscience.2007.09.013 PMC2189738 17936518

[ejn70386-bib-0078] West, A. R. , and M. P. Galloway . 1997. “Inhibition of Glutamate Reuptake Potentiates Endogenous Nitric Oxide‐Facilitated Dopamine Efflux in the Rat Striatum: An In Vivo Microdialysis Study.” Neuroscience Letters 230: 21–24. 9259454 10.1016/s0304-3940(97)00465-5

[ejn70386-bib-0098] West, A. R. , M. P. Galloway , and A. A. Grace . 2002. “Regulation of Striatal Dopamine Neurotransmission by Nitric Oxide: Effector Pathways and Signaling Mechanisms.” Synapse 44, no. 4: 227–245. 10.1002/syn.10076. 11984858

[ejn70386-bib-0079] Wiley, J. L. 1998. “Nitric Oxide Synthase Inhibitors Attenuate Phencyclidine‐Induced Disruption of Prepulse Inhibition.” Neuropsychopharmacology 19, no. 1: 86–94. 9608580 10.1016/S0893-133X(98)00008-6

[ejn70386-bib-0080] Yanahashi, S. , K. Hashimoto , K. Hattori , S. Yuasa , and M. Iyo . 2004. “Role of NMDA Receptor Subtypes in the Induction of Catalepsy and Increase in Fos Protein Expression After Administration of Haloperidol.” Brain Research 1011, no. 1: 84–93. 10.1016/j.brainres.2003.12.059. 15140647

[ejn70386-bib-0081] Zarrindast, M. R. , M. Modabber , and M. Sabetkasai . 1993. “Influences of Different Adenosine Receptor Subtypes on Catalepsy in Mice.” Psychopharmacology 113: 257–261. 7855191 10.1007/BF02245707

[ejn70386-bib-0094] Zhang, J. , C. G. Abdallah , J. Wang , et al. 2012. “Upregulation of Adenosine A2A Receptors Induced by Atypical Antipsychotics and Its Correlation with Sensory Gating in Schizophrenia Patients.” Psychiatry Research 200, no. 2‐3: 126–132. 10.1016/j.psychres.2012.04.021. 22705363 PMC3449024

[ejn70386-bib-0082] Zhang, L. , and J. Zhao . 2014. “Profile of Minocycline and Its Potential in the Treatment of Schizophrenia.” Neuropsychiatric Disease and Treatment 10: 1103–1111. 24971013 10.2147/NDT.S64236 PMC4069141

