# Supplementary material for: Striatal Nitric Oxide Activity Better Predicts Motor Disability Than Proto‐Oncogenes
Source: Eur J Neurosci. 2026 Jan 13;63(1):e70386. doi: 10.1111/ejn.70386 (PMC12797185; doi:10.1111/ejn.70386)
Supplement: Supplementary file 1 — Table S1: Behavioral ANOVA results. Table S2: Histochemistry ANOVA results. [file EJN-63-0-s001.pdf]

Supplementary Table 1: Behavioral ANOVA results

	Catalepsy		
Drug	**Test**	**F (df)**	**p-value**
**Haloperidol**	Time	F(4,64) = 7.935	p < 0.002
**Haloperidol**	Treatment	F(3,16) = 241.4	p < 0.001
**Haloperidol**	Time × Treatment	F(12,64) = 21.227	p < 0.001
**Metoclopramide**	Time	F(4,80) = 16.454	p < 0.001
**Metoclopramide**	Treatment	F(4,20) = 60.92	p < 0.001
**Metoclopramide**	Time × Treatment	F(16,80) = 5.043	p < 0.001
**L-NOARG**	Time	F(4,64) = 12.287	p < 0.001
**L-NOARG**	Treatment	F(3,16) = 12.37	p < 0.001
	**Rota-Rod Test**		
Drug	**Test**	**F (df)**	**p-value**
**Haloperidol**	Treatment	F(3,16) = 34.10	p < 0.001
**Metoclopramide**	Treatment	F(4,20) = 5.079	p = 0.002

Supplementary Table 2: Histochemistry ANOVA results

		c-Fos		
Drug	**Dose**	**Region**	**F (df)**	**p-value**
**Hal**	0.1 and 1 mg/kg	DL	F(3,16) = 42.856	p < 0.001
**Hal**	1 mg/kg	DM	F(3,16) = 9.793	p < 0.001
**Hal**	0.1 and 1 mg/kg	VL	F(3,16) = 19.038	p < 0.001
**Hal**	1 mg/kg	NAc Shell	F(3,16) = 10.283	p < 0.001
**Hal**	1 mg/kg	NAc Core	F(3,16) = 4.019	p = 0.026
**MCP**	5, 8, 45 mg/kg	DL	F(4,20) = 14.30	p < 0.001
**MCP**	1 mg/kg	DM	F(4,20) = 3.207	p = 0.035
**MCP**	45 mg/kg	VL	F(4,20) = 6.304	p = 0.002
**L-NOARG**	30, 45 mg/kg	DL	F(3,16) = 4.562	p = 0.017
**L-NOARG**	30, 45 mg/kg	DM	F(3,16) = 9.673	p < 0.001
**L-NOARG**	30, 45 mg/kg	VL	F(3,16) = 8.413	p = 0.001

**L-NOARG**	30, 45 mg/kg	Shell	F(3,16) = 7.644	p = 0.002
**L-NOARG**	30 mg/kg	Core	F(3,16) = 12.288	p < 0.001
		**nNos**		
Drug	Dose	Region	F (df)	p-value
**Hal**	0.1 and 0.5 mg/kg	DL	F(3,16) = 7.477	p = 0.002
**Hal**	0.1 mg/kg	DM	F(3,16) = 4.308	p = 0.021
**MCP**	8 mg/kg	DL	F(4,20) = 5.275	p = 0.005
**MCP**	8 mg/kg	VL	F(4,20) = 4.780	p = 0.007
**L-NOARG**	15 mg/kg	DL	F(3,16) = 3.609	p = 0.037
**L-NOARG**	30 mg/kg	DM	F(3,16) = 6.336	p = 0.005
		**NADPH-d**		
Drug	Dose	Region	F (df)	p-value
**Hal**	0.1,0.5 and 1 mg/kg	DL	F(3,16) = 12.341	p < 0.001
**Hal**	0.1 and 0.5 mg/kg	DM	F(3,16) = 11.091	p < 0.001
**Hal**	0.1 and 0.5 mg/kg	VL	F(3,16) = 8.413	p = 0.001
**MCP**	8 mg/kg	DL	F(4,20) = 15.20	p < 0.001
**MCP**	8 and 45 mg/kg	DM	F(4,20) = 10.56	p < 0.001
**MCP**	8 mg/kg	VL	F(4,20) = 3.915	p = 0.017
**L-NOARG**	15,30 and 45 mg/kg	DL	F(3,16) = 9.467	p < 0.001
**L-NOARG**	15,30 and 45 mg/kg	DM	F(3,16) = 15.748	p < 0.001
**L-NOARG**	45 mg/kg	VL	F(3,16) = 4.505	p = 0.018
**L-NOARG**	30 mg/kg	NAc Shell	F(3,16) = 5.017	p = 0.012
**L-NOARG**	15 and 45 mg/kg	NAc Core	F(3,16) = 4.350	p = 0.020
